# Anti-SARS-CoV-2 activities of tanshinone IIA, carnosic acid, rosmarinic acid, salvianolic acid, baicalein, and glycyrrhetinic acid between computational and *in vitro* insights†

**DOI:** 10.1039/d1ra05268c

**Published:** 2021-09-01

**Authors:** Dalia Elebeedy, Walid F. Elkhatib, Ahmed Kandeil, Aml Ghanem, Omnia Kutkat, Radwan Alnajjar, Marwa A. Saleh, Ahmed I. Abd El Maksoud, Ingy Badawy, Ahmed A. Al-Karmalawy

**Affiliations:** College of Biotechnology, Misr University for Science and Technology (MUST) 6th of October City Egypt; Microbiology and Immunology Department, Faculty of Pharmacy, Ain Shams University, African Union Organization St. Abbassia Cairo 11566 Egypt; Department of Microbiology & Immunology, Faculty of Pharmacy, Galala University New Galala city, Suez Egypt; Center of Scientific Excellence for Influenza Viruses, National Research Centre Giza 12622 Egypt; Department of Molecular Biology, Genetic Engineering and Biotechnology Research Institute, University of Sadat City Sadat City Egypt; Department of Chemistry, Faculty of Science, University of Benghazi Benghazi Libya; Department of Chemistry, University of Cape Town Rondebosch 7701 South Africa; Department of Pharmaceutical Organic Chemistry, Faculty of Pharmacy (Girls), Al-Azhar University Nasr City Cairo Egypt; Industrial Biotechnology Department, Genetic Engineering and Biotechnology Research Institute, University of Sadat City Sadat City Egypt; Department of Pharmaceutical Medicinal Chemistry, Faculty of Pharmacy, Horus University-Egypt New Damietta 34518 Egypt akarmalawy@horus.edu.eg

## Abstract

Six compounds namely, tanshinone IIA (1), carnosic acid (2), rosmarinic acid (3), salvianolic acid B (4), baicalein (5), and glycyrrhetinic acid (6) were screened for their anti-SARS-CoV-2 activities against both the spike (S) and main protease (Mpro) receptors using molecular docking studies. Molecular docking recommended the superior affinities of both salvianolic acid B (4) and glycyrrhetinic acid (6) as the common results from the previously published computational articles. On the other hand, their actual anti-SARS-CoV-2 activities were tested *in vitro* using plaque reduction assay to calculate their IC_50_ values after measuring their CC_50_ values using MTT assay on Vero E6 cells. Surprisingly, tanshinone IIA (1) was the most promising member with IC_50_ equals 4.08 ng μl^−1^. Also, both carnosic acid (2) and rosmarinic acid (3) showed promising IC_50_ values of 15.37 and 25.47 ng μl^−1^, respectively. However, salvianolic acid (4) showed a weak anti-SARS-CoV-2 activity with an IC_50_ value equals 58.29 ng μl^−1^. Furthermore, molecular dynamics simulations for 100 ns were performed for the most active compound from the computational point of view (salvianolic acid 4), besides, the most active one biologically (tanshinone IIA 1) on both the S and Mpro complexes of them (four different molecular dynamics processes) to confirm the docking results and give more insights regarding the stability of both compounds inside the SARS-CoV-2 mentioned receptors, respectively. Also, to understand the mechanism of action for the tested compounds towards SARS-CoV-2 inhibition it was necessary to examine the mode of action for the most two promising compounds, tanshinone IIA (1) and carnosic acid (2). Both compounds (1 and 2) showed very promising virucidal activity with a most prominent inhibitory effect on viral adsorption rather than its replication. This recommended the predicted activity of the two compounds against the S protein of SARS-CoV-2 rather than its Mpro protein. Our results could be very promising to rearrange the previously mentioned compounds based on their actual inhibitory activities towards SARS-CoV-2 and to search for the reasons behind the great differences between their *in silico* and *in vitro* results against SARS-CoV-2. Finally, we recommend further advanced preclinical and clinical studies especially for tanshinone IIA (1) to be rapidly applied in COVID-19 management either alone or in combination with carnosic acid (2), rosmarinic acid (3), and/or salvianolic acid (4).

## Abstract 2

Tanshinone IIA shows the most promising anti-SARS-CoV-2 biological activity: molecular docking, molecular dynamics, *in vitro*, and SAR studies.

## Graphical abstract

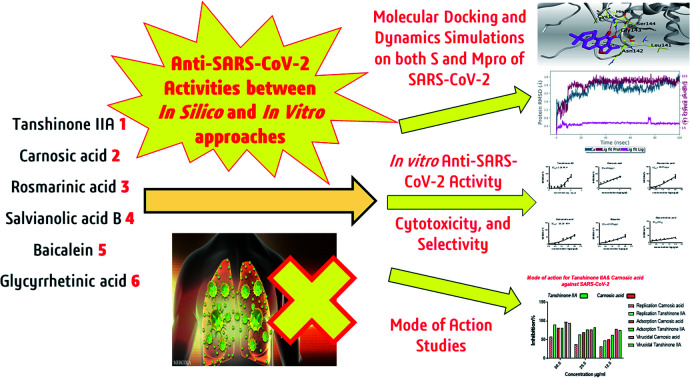

## 1. Introduction

The coronavirus outbreak came to light in December 2019 and WHO has declared it a pandemic.^1^ It has been named coronavirus disease 19 (COVID-19), which is known for its high infectivity and pathogenicity, and its causative virus was named Severe Acute Respiratory Syndrome Coronavirus-2 (SARS-CoV-2).^2^

Coronaviruses generally infect the lower respiratory tract and their spike proteins are critical for host cell entry,^3^ because of highly mutated spikes there is an urgent need for safe and effective drugs by finding a new broad-spectrum anti-coronavirus candidate, such as spike protein inhibitors that halting the fusion of the spike (S) protein of coronaviruses and angiotensin-converting enzyme 2 (ACE2) of the host.^4^ Scientists have been screened for new compounds from medicinal plants to avert the COVID-19 global crisis,^5^ it could be through halting the activity of enzymes associated with the virus replication cycle, including 3C-like protease (3CLpro) and papain-like protease (PLpro), and also inhibit cellular signaling pathways to prevent COVID-19 or at least to relieve its deadly symptoms.^6^ Besides, angiotensin II receptor blockers to inhibit the SARS-CoV-2 main protease (Mpro) is an important hotspot for the treatment.^7^

On the other hand, an acute respiratory distress syndrome (ARDS) may appear in SARS-CoV infected patients, especially in patients with severe COVID-19 infection. Cytokine storm has been found as an immunological response to viral infection. So, a significant increase in cytokines such as IL-2, IL-7, IL-10, monocyte chemoattractant protein-1 (MCP1), granulocyte colony-stimulating factor (GSCF), macrophage inflammatory protein 1A (MIP1A), IFN-γ-induced protein-10 (IP10), and tumor necrosis factor-α (TNF-α) was characteristic to severe COVID-19 patients which may have hugely damaging effects. Therefore, the administration of effective anti-inflammatory drugs is a crucial treatment strategy to save patients' lives and reduce the mortality rate.^8^

However, alternative natural compounds are crucial to human health for their safe therapeutic actions since ancient times.^9,10^ They have a wide application in pharmaceutical industries, such as inflammation, cancer, oxidative process, and viral infections drugs.^11^ Many antiviral bioproducts have already been described against hepatitis B (HBV), influenza virus, human immunodeficiency virus (HIV), and coronavirus.^12^

We find that alternative natural products are an important source that can be used as a basis for new drug development targeting these viruses. Therefore, our research aims to administrate some potential compounds from plant sources that possess an antiviral alternative approach against SARS-CoV-2.

Tanshinone IIA (TSN) is the main active constituent of *Salvia miltiorrhiza*, which is traditional Chinese medicine.^13^ It is a highly anti-oxidant compound, and it reduces liver injury significantly and reduces the inflammatory cytokines, including IL-2, IL-4, INF-γ, and TNF-α.^14^ It could also attenuate traumatic brain injury by inhibiting oxidative stress and apoptosis as proposed mechanisms of its action.^15^ It displayed a protective effect against lung injury and it has an anti-pulmonary fibrosis effect.^16^ It can also inhibit the cytokines and platelets by an aspirin-like effect and so decrease the inflammation damage of vessels in patients with immune vasculitis.^17^ It decreases the expression of transforming growth factor-beta 1 superfamily of cytokines (TGF-β1) and reversed ACE-2 and angiotensin (ANG) (1–7) production in rat lungs.^18^

Carnosic acid (CA) is a diterpene found in many plants including rosemary and sage. It has been known for its antioxidative and antimicrobial properties, and it is a safe compound that can be applied within the food and cosmetics industries.^19^ It showed anti-SARS-CoV-2 activity due to its higher binding affinity to the inhibitory site of the Mpro.^20^ It decreases the levels of TNF-α, IL-6, and IL-1β through inhibiting the nuclear factor kappa-light-chain-enhancer of activated B cells (NF-κB) pathway which is important for the activation of neutrophils and responsible for the inflammatory responses of acute lung injury.^21^

Rosmarinic acid is a phenolic compound that was found in many plants, like those of the Boraginaceae and Lamiaceae families.^7^ It displays a general anti-oxidant and anti-inflammatory potentiality, and it serves as an anti-viral agent by its binding affinity to SARS-CoV-2 viral protein targets. Furthermore, it could act as a nutritional supplement that improves the immunity against COVID-19.^22^ It was found to inhibit interleukin-6 (IL-6) secretion, decrease total immunoglobulin E (IgE) concentrations, and significantly alleviate oxidative lung damage and airway inflammation during asthma.^23^ It is the potential to combat acute asthmatic attacks and reduce allergic airway reactivity in long-term use.^24^

Salvianolic acid B (Sal B) is a natural phenolic acid extracted from *Salvia miltiorrhiza* root, widely used in traditional Chinese medicine, and known for its anti-oxidant potentiality.^25^ It exerts significant protective activity against lung injury and pulmonary fibrosis throughout decreasing TNF-α, IL-6, and IL-17.^26^ It can affect the Ca^2+^ aggregation and reduce oxidative damage.^27^ Sal B has a pivotal interaction with Cys145, Gly166, Gln189, His41, Thr190, Thr24, Gly143, and other residues of the active site of SARS-CoV-2.^28,29^

Baicalein is an isolated flavonoid from the roots of *Scutellaria baicalensis* which has a broad anti-viral effect.^30^ It was recorded to improve respiratory function, inhibit inflammatory cell infiltration in the lung, and decrease the levels of IL-1β and TNF-α in serum^31^ as well as can reduce the intercellular reactive oxygen species (ROS) that could inhibit the cell damage caused by SARS-CoV-2. It halted the replication of coronaviruses and relieved the lung tissue lesions in *h*ACE2 transgenic mice.^32^ Baicalein inhibits oxidative phosphorylation (OXPHOS) which is a novel mode of action for the antiviral drug development through targeting the mitochondrial OXPHOS in an mPTP dependent manner, a recently defined OXPHOS component playing critical roles in mitochondrial membrane potential (MMP) regulation.^33^

Glycyrrhetinic acid is the main active constituent of liquorice root which has been traditionally prescribed for treating asthma, dry cough, and other pectoral diseases. It could alleviate bronchitis, acting as anti-inflammatory and antioxidant, and stimulate the endogenous production of interferons which have very good potentiality against different viruses,^34^ including influenza virus, hemagglutinin type 5 and neuraminidase type 1 (Avian Influenza A) (H5N1), and SARS-associated human and animal coronaviruses.^35,36^ It has been recorded recently for its binding with ACE2 to prevent SARS-CoV-2 infection.^36^ Traditional Chinese Medicine (TCM) treatments for SARS-CoV-2 pneumonia were recommended by the National Health Commission of China, and liquorice root was one of the commonly used TCM herbs, while the FDA-approved, glycyrrhizin as a general tonic, antioxidant, cell-protective, and immune stimulant,^37^ by reducing TNF-α^38^ and downregulating other proinflammatory cytokines, in addition to preventing reactive oxygen species (ROS) accumulation, inhibiting thrombin, and inducing endogenous interferon.^39^

However, molecular docking is one of the most important and helpful methods of computational drug design for finding new drug members.^40^ Therefore, newer drug candidates could be introduced according to their chemical nature and the recommended target receptor, saving effort, time and cost.^23^ Moreover, molecular dynamics simulations are useful for analyzing the physical movements of atoms and molecules within the system by allowing them to interact freely for a certain time in similar physiological conditions.^41^

**Fig. 1:**
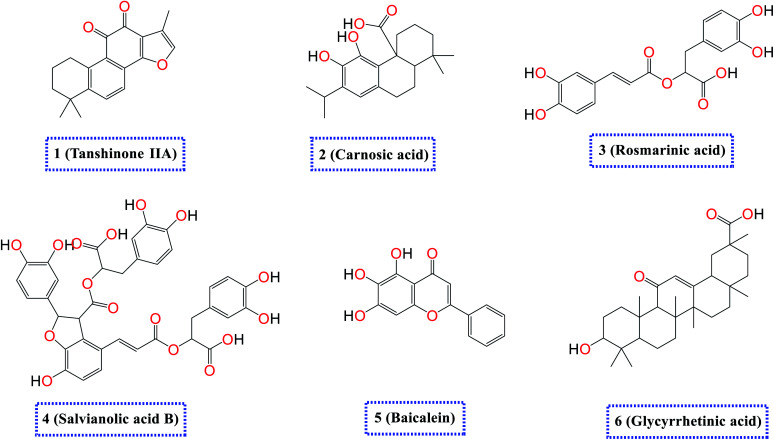
Chemical structures of the selected natural compounds (tanshinone IIA 1, carnosic acid 2, rosmarinic acid 3, salvianolic acid B 4, baicalein 5, and glycyrrhetinic acid 6).

Accordingly, as an extension to our previous work targeting SARS-CoV-2,^2,7,29,42–48^ and taking into consideration the crucial role of both SARS-CoV-2 spike (S) and main protease (Mpro) proteins for the viral activity, pathogenicity, and replication besides the above-mentioned reported antiviral effects of the selected natural compounds (1–6) depicted in (Fig. 1), we examined their antiviral effects against both the S and the Mpro of SARS-CoV-2 *via* molecular docking (PDB ID 6VW1 (ref. 30) and 6LU7,^49^ respectively) and confirmed it through deep *in vitro* antiviral studies against SARS-CoV-2 in VERO-E6 cells. Furthermore, we examined the mode of antiviral action of the most two promising members of the tested compounds.

## 2. Experimental

### 2.1. Docking studies

The selected natural compounds (1–6) were examined for their binding potentials towards two important pathogenic factors of SARS-CoV-2 (spike (S) and main protease (Mpro) proteins) using N3, the natural inhibitor of the main protease, as a reference standard in case of the main protease *via* molecular docking using MOE 2019 suite.^50^

#### 2.1.1. Preparation of the examined natural compounds

The chemical structures of tanshinone IIA (1), carnosic acid (2), rosmarinic acid (3), salvianolic acid B (4), baicalein (5), and glycyrrhetinic acid (6) were downloaded from the PubChem database and then prepared for docking as the default procedure.^51^ They were subjected to energy minimization and partial charges calculation processes as well.^52^ Then, the prepared compounds (1–6) were inserted in two different databases, the first one containing only the tested compounds and the second containing the tested compounds besides the main protease co-crystallized inhibitor (N3), and saved as two separate MDB files for docking against spike protein and main protease pockets, respectively.

#### 2.1.2. Preparation of SARS-CoV-2 spike and main protease target pockets

The X-ray structures of both SARS-CoV-2 spike (S) and main protease (Mpro) proteins were extracted from the protein data bank (PDB codes 6VW1 (ref. 30) and 6LU7,^49^ respectively). They were protonated, corrected, and energy minimized to be prepared for docking processes as discussed in detail previously.^53^

#### 2.1.3. Docking of the prepared compounds (1–6) to the viral spike and main protease pockets

At the start, to validate the docking process of the MOE program and ensure its accuracy, we performed a redocking process for the N3 co-crystallized inhibitor of Mpro enzyme, and a valid performance of the program was confirmed by obtaining a low value of RMSD (1.23 Å).^54,55^

Then, two separate docking processes were carried out using the above-mentioned two databases towards spike (S) and main protease (Mpro) pockets, respectively. The general docking protocol was applied according to the default methodology described in detail earlier^51^ to choose poses with the best binding scores, RMSD values, and amino acid interactions. The applied methodology is based on uploading the file of the prepared active site in each case, besides adjusting the program specifications as follows (the docking site was selected to be as dummy atoms, the placement methodology was triangle matcher, and the scoring methodology was London dG). Also, the rigid receptor was selected as the refinement methodology and GBVI/WSA dG as the scoring methodology for selection of the best poses as discussed above.^56,57^

### 2.2. Molecular dynamics (MD) simulations

MD simulations were conducted using the Desmond package (Schrödinger LLC).^58^ and the Molecular Mechanics Generalized Born Surface Area (MM-GBSA) energies for all complexes were calculated using the thermal_mmgbsa.py python script provided by Schrödinger. Details of the molecular dynamics simulation are provided in ESI 1 and 2.†

### 2.3. *In vitro* studies

#### 2.3.1. MTT cytotoxicity assay

This assay is to know concentrations of compounds that cause toxicity to 50% of the cells (CC_50_). The tested compounds were dissolved in ddH_2_O with 10% DMSO and diluted with DMEM during working. The cytotoxic activity was tested in VERO-E6 cells due to this type from cells suitable for propagation type of virus which will be used in other experiments, by using the MTT method with minor modifications. Collectively, the cells were cultivated in 96 well-plates and incubated for 24 h in 5% CO_2_ at 37 °C. 24 h later, compounds were diluted with DMEM in HA plate in triplicates. Then, the diluted compounds were added to the previously prepared cells after washing 2 times using sterile 1× phosphate buffer saline (PBS). After 24 h incubation in 5% CO_2_ at 37 °C, the supernatant was removed, and then cell monolayers were washed for 3 times with sterile 1× PBS and MTT solution was poured into each well (20 μl of 5 mg ml^−1^ stock solution) and incubated for 4 h at 37 °C. 200 μl of acidified isopropanol was used to dissolve the formed formazan crystals. Finally, the absorbance of formazan solutions was recorded using a multi-well plate reader at *λ*_max_ 540 nm with 620 nm as a reference wavelength. The % of cytotoxicity compared to the untreated control cells was determined using the following equation:
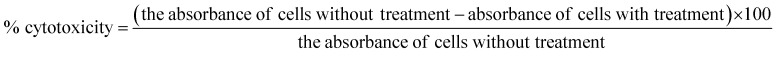


#### 2.3.2. Plaque reduction assay

This assay was performed in a six-well plate according to the method of (Hayden *et al.*, 1980)^59^ where Vero E6 cells (105 cells per ml) were cultivated at 37 °C for 24 h. SARS-CoV-2 (hCoV-19/Egypt/NRC-03/2020, accession number on GSAID: EPI_ISL_430820) was diluted to give 10^3^ PFU per well, this dilution was prepared according to plaque assay test result, and mixed with the safe concentrations of the tested compounds, and incubated at 37 °C for 1 h before being added to the cells. The cells were inoculated with (100 μl per well) virus with the tested compounds, after removal of the growth medium from the cell culture plates. 1 h later of contact to allow for virus adsorption, the supernatant was removed and 3 ml of DMEM was added containing 2% agarose. The tested compounds were added onto the cell over layers, plates were left for 3–4 days to solidify and incubated at 37 °C till the formation of viral plaques. 10% Formalin was added for 2 h then washed with H_2_O and plates were stained with 0.1% crystal violet in distilled H_2_O. Wells containing untreated viruses only as control were included as cell control. Finally, the plaques were counted and % reduction in plaques formation compared to control wells was recorded according to: % inhibition = viral count (untreated) − viral count (treated)/viral count (untreated) × 100.

#### 2.3.3. Inhibitory concentration 50 (IC_50_) determination

2.4 × 104 Vero-E6 cells were distributed in 96-well tissue culture plates and incubated overnight in 5% CO_2_ at 37 °C. Then, the cell monolayers were washed with 1× PBS for one time and subjected to serial dilutions of the tested compounds mixed with fixed dilution from the virus (hCoV-19/Egypt/NRC-03/2020 (accession number on GSAID: EPI_ISL_430820)) according to TCID50 test and incubated for 1 h at RT before adding to the cells, after first incubation 100 μl of DMEM mixture consists of varying concentrations of the test samples and virus were also added to the cell monolayers to start the second incubation for 72 h at 37 °C in a 5% CO_2_ incubator, 100 μl of 4% paraformaldehyde was added for 2 h for cell fixation and staining using 50 μl of 0.1% crystal violet in distilled H_2_O was done for 15 min at RT. 100 μl of absolute CH_3_OH was used to dissolve the crystal violet dye per well and the produced color optical density was measured at 570 nm using Anthos Zenyth 200rt plate reader.^60^ The concentration of compounds required to reduce the infectivity of the virus by 50% relative to the virus control (IC_50_) was calculated.

#### 2.3.4. Mechanism of action studies

All possible mechanisms for HCoV-19/Egypt/NRC-1/2020 virus inhibition by the most promising two compounds (tanshinone IIA 1 and carnosic acid 2) were tested as follow:

##### 2.3.4.1. Viral replication^61,62^

This assay was performed using Vero E6 cells which were cultivated at 37 °C in 5% CO_2_ for 24 h in a 6 well plate (105 cell per ml). HCoV-19/Egypt/NRC-1/2020 virus was diluted to obtain 10^3^ PFU per well, added directly to the cells, and incubated for 1 h at 37 °C. Then, the cells were washed 3 times using 1× PBS to remove the excess viral particles following viral adsorption. 100 μl of the tested compounds with safe different concentrations with 300 μl infection medium were incubated for 1 h. Then, 3 ml of 2× DMEM medium containing agarose (2%) was added to the cell monolayer. Plates were incubated at 37 °C and left to solidify till the appearance of viral plaques. 10% Formaldehyde was used to fix the cell monolayers for 2 h which were then stained with crystal violet. Control wells with Vero E6 cells were incubated with the virus and plaques were counted and a % reduction in plaques formation compared to the control wells was recorded as previously mentioned.

##### 2.3.4.2. Viral adsorption^63^

Vero E6 cells were seeded in a 6 well plate (105 cell per ml) for 24 h at 37 °C with 5% CO_2_. 100 μl of compounds were added with safe different concentrations with 300 μl infection medium and incubated with the cells at 4 °C for 1 h. Washing cells 3 successive times with 1× PBS to remove the unabsorbed drug, then HCoV-19/Egypt/NRC-1/2020 virus was diluted to give 10^3^ PFU per well and co-incubated with the pretreated cells for 1 h followed by adding 3 ml 2× DMEM containing agarose (2%) after the supernatant removal. After the solidification of plates, they were incubated at 37 °C to allow the formation of viral plaques. Finally, the plates were fixed and stained as previously mentioned to calculate the % reduction in plaque formation compared to control wells of cells directly infected with the virus.

##### 2.3.4.3. Virucidal^64^

The following assay in a 6 well plate was carried out where Vero E6 cells were seeded (105 cell per ml) at 37 °C with 5% CO_2_ for 24 h. HCoV-19/Egypt/NRC-1/2020 virus diluted to obtain 10^3^ PFU per well and 100 μl from the virus was added to 100 μl of compounds with safe different concentrations. After 1 h incubation, the mixtures were added to the cells monolayer. Further 1 h of contact time, the supernatant was removed followed by the addition of 3 ml 2× DMEM supplemented with agarose (2%). As discussed before and to allow the formation of viral plaques, the plates were kept to solidify and then incubated at 37 °C in presence of 5% CO_2_. Fixation and staining of the plates as mentioned above to calculate % reduction in plaques formation compared to the control wells.

## 3. Results and discussions

### 3.1. Docking studies

Molecular docking of the examined natural compounds (1–6) into the spike (S) active site of COVID-19 and its main protease (Mpro) active site together with the N3 natural inhibitor (7) (in case of Mpro docking) were done. The descending binding order for the examined natural compounds based on the score values against the spike (S) protein of SARS-CoV-2 was as follows: salvianolic acid (4) > glycyrrhetinic acid (6) > rosmarinic acid (3) > carnosic acid (2) > tanshinone IIA (1) > baicalein (5). However, their descending binding order against the Mpro was: salvianolic acid (4) > glycyrrhetinic acid (6) > tanshinone IIA (1) > rosmarinic acid (3) > carnosic acid (2) > baicalein (5).

The scores and RMSD values of the examined natural compounds, besides their different amino acid interactions inside the S and Mpro pockets of SARS-CoV-2 were depicted in Table 1.

**Table 1:** Binding scores, RMSD values, and amino acid interactions of the tested compounds (1–6), into the binding sites of SARS-CoV-2 spike (S) and main protease (Mpro)

Compound	Pocket	Score^a^	RMSD_refine^b^	Interactions	Distance Å
1	S	−5.80	1.04	His195/pi-H	3.60
	Mpro	−6.67	1.75	Cys145/H-acceptor	3.02
				His163/H-acceptor	3.22
				Asn142/pi-H	3.81
				Asn142/pi-H	4.54
2	S	−6.12	1.18	Asn194/H-donor	3.07
				Asn194/H-donor	3.52
	Mpro	−6.11	1.45	Cys145/H-acceptor	3.13
				Met165/H-acceptor	3.30
				Asn142/pi-H	4.14
3	S	−6.16	1.29	Gln86/H-donor	3.02
				Gln81/H-donor	3.34
	Mpro	−6.63	1.44	Glu166/H-acceptor	3.06
				Met165/H-donor	4.02
				Asn142/pi-H	4.09
				Gln189/pi-H	4.18
4	S	−7.85	1.69	Gln101/H-donor	2.75
				Gln98/H-donor	2.95
				Asn103/H-donor	3.01
				Asn194/H-donor	3.22
	Mpro	−9.23	2.19	His163/H-acceptor	3.05
				Glu166/H-donor	3.39
5	S	−5.73	0.85	Gln81/H-donor	2.96
				Glu81/pi-H	3.81
				Gln102/pi-H	4.07
				Gln101/pi-H	4.50
	Mpro	−5.83	1.07	Leu141/H-donor	2.81
				Glu166/H-acceptor	3.24
6	S	−6.90	2.14	Gln81/H-donor	2.96
	Mpro	−6.77	1.59	Ser46/H-acceptor	2.84
				Glu166/H-acceptor	2.98
N3, 7	Mpro	−10.70	2.30	Leu141/H-donor	2.85
				Gln189/H-donor	2.87
				Thr190/H-donor	3.04
				Glu166/H-acceptor	3.10
				Glu166/H-donor	3.15
				His163/H-acceptor	3.43
				His164/H-donor	3.54
				Thr25/pi-H	4.09
				Thr26/pi-H	4.15

a S: score of a compound inside the protein binding pocket (kcal mol^−1^).

b RMSD_refine: root mean squared deviation after and before refinement between the predicted pose and the crystal structure, respectively.

Analyzing the aforementioned docking results (Tables 1 and 2) of our tested compounds (1–6) towards both the S and Mpro pockets of SARS-CoV-2, we can conclude the following:

**Table 2:** 3D pictures representing the binding interactions and positioning of the tested natural compounds (1–6) inside both S and Mpro pockets of the SARS-CoV-2, besides the N3 inhibitor of Mpro (redocked, 7)^a^

Comp.	Pocket	3D interactions	3D protein positioning
1	S	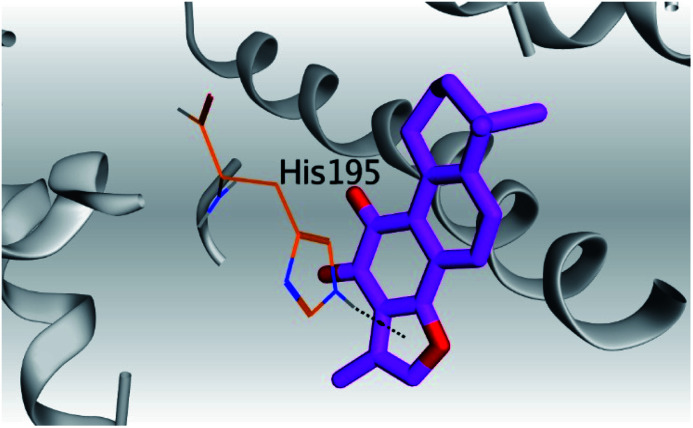	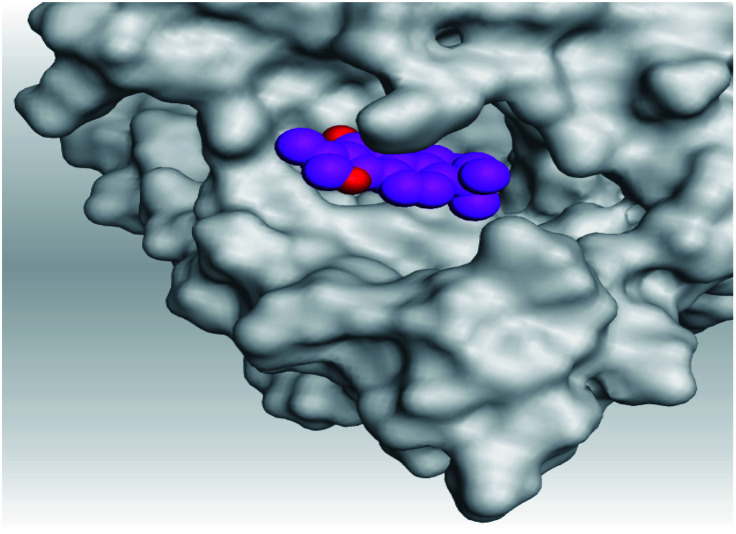
	Mpro	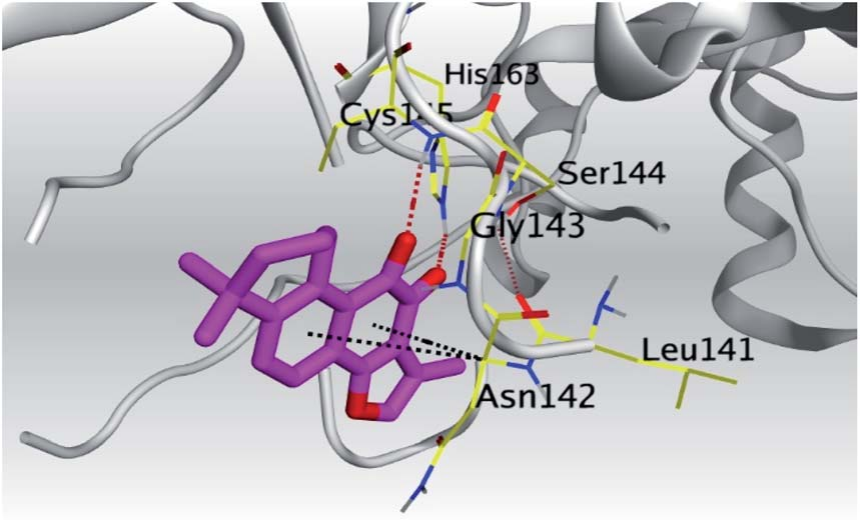	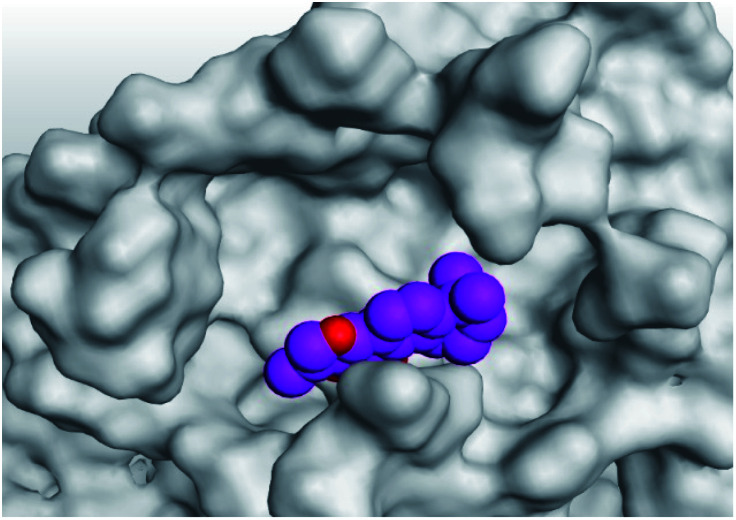
2	S	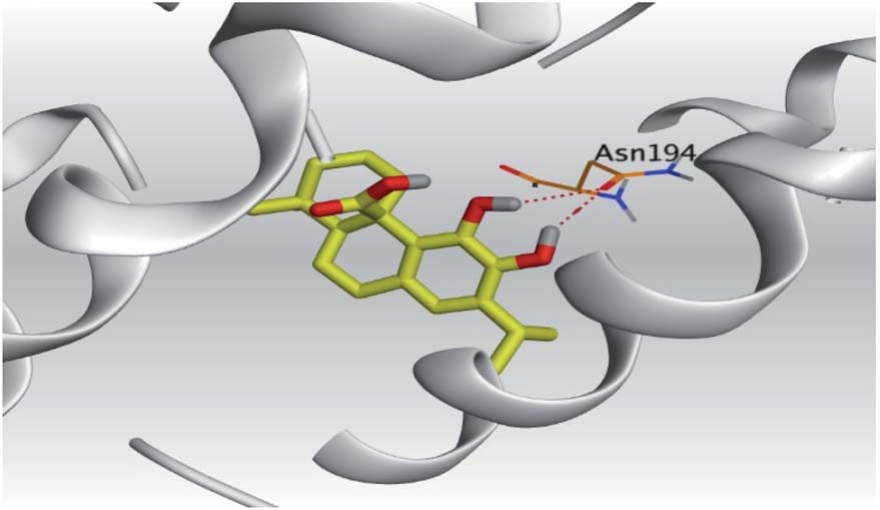	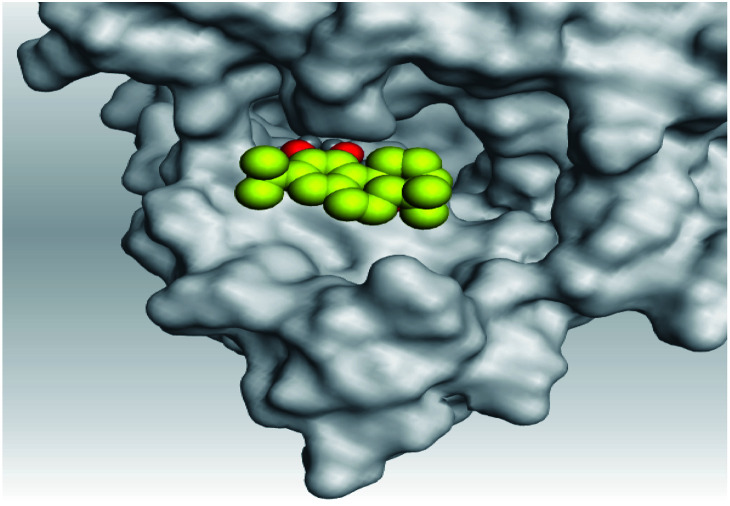
	Mpro	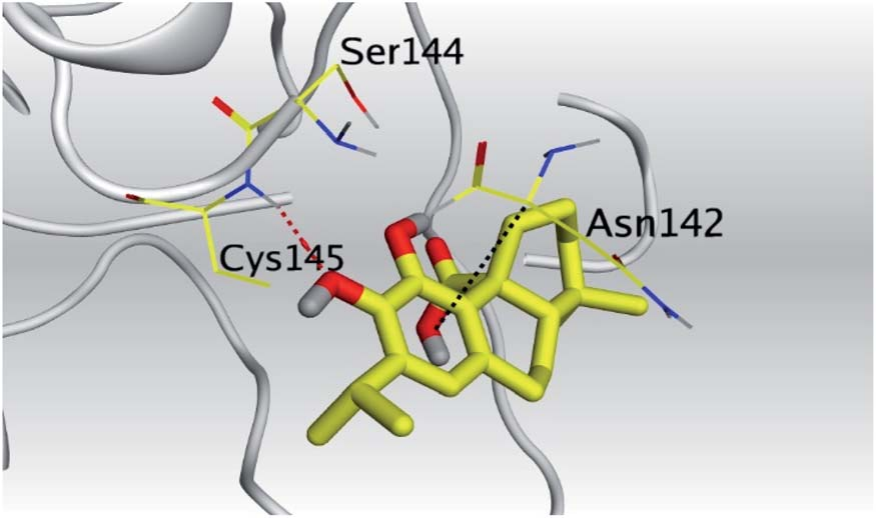	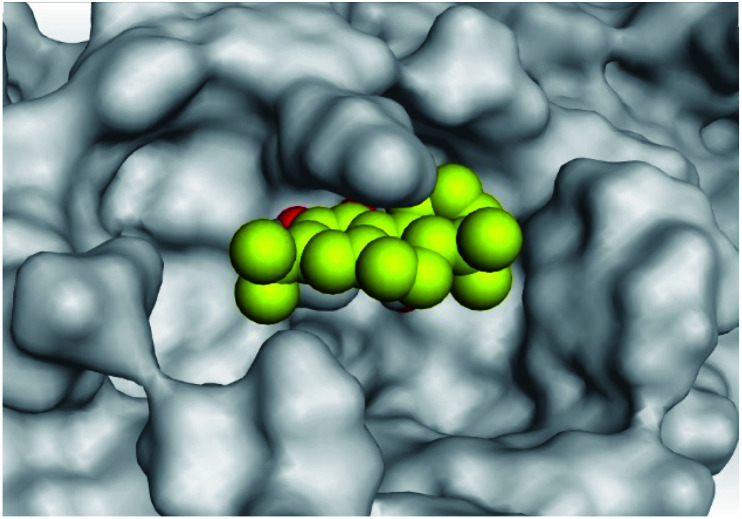
3	S	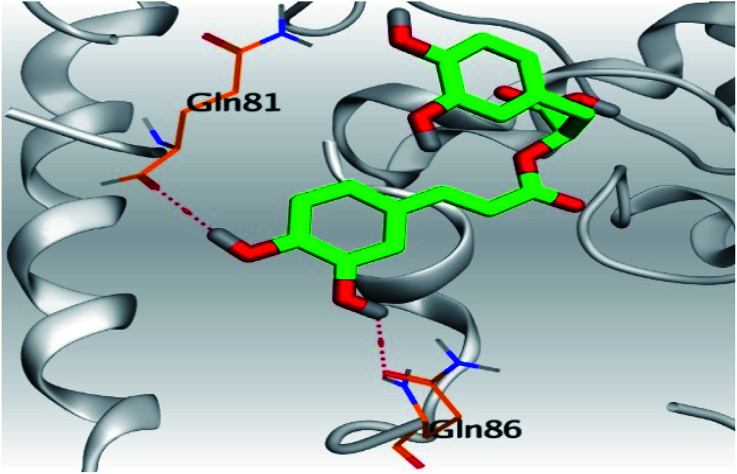	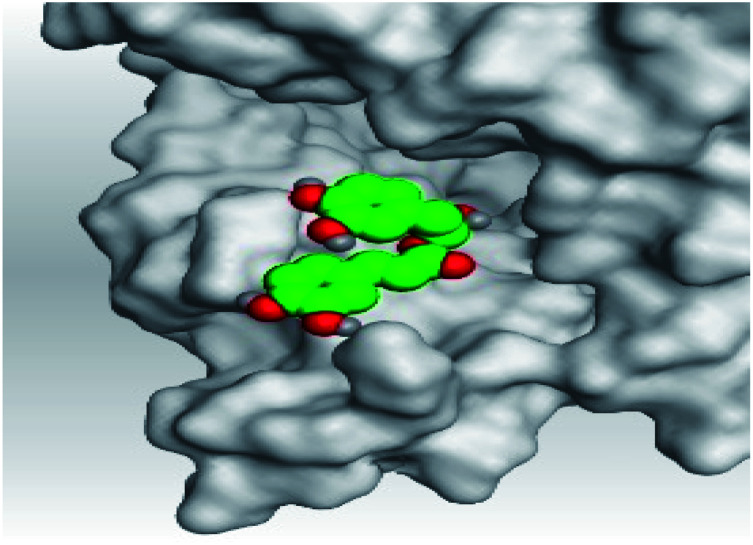
	Mpro	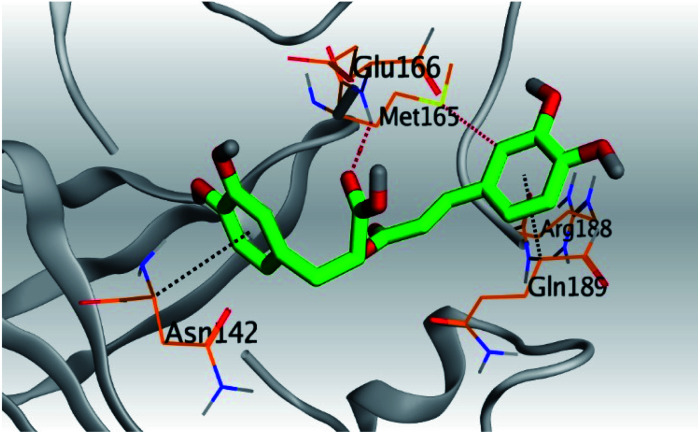	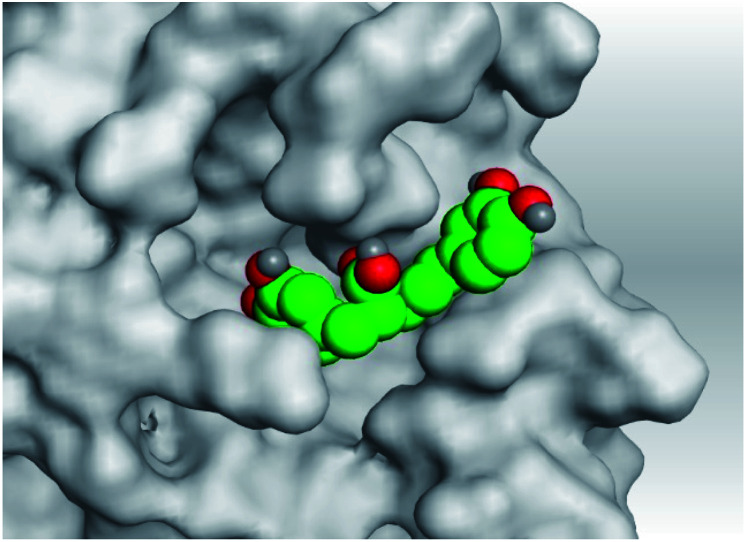
4	S	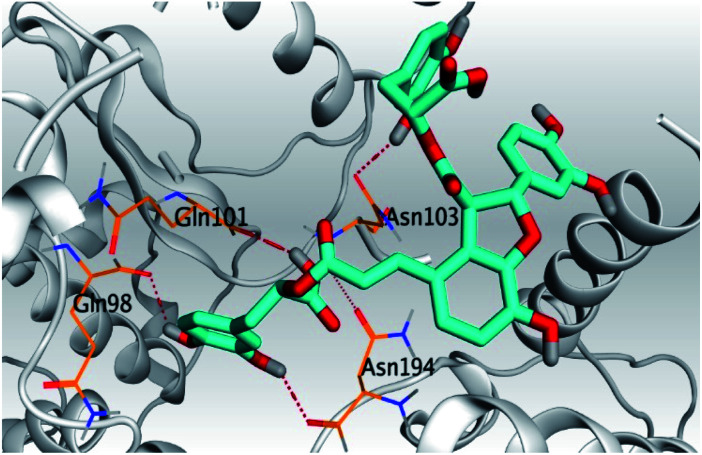	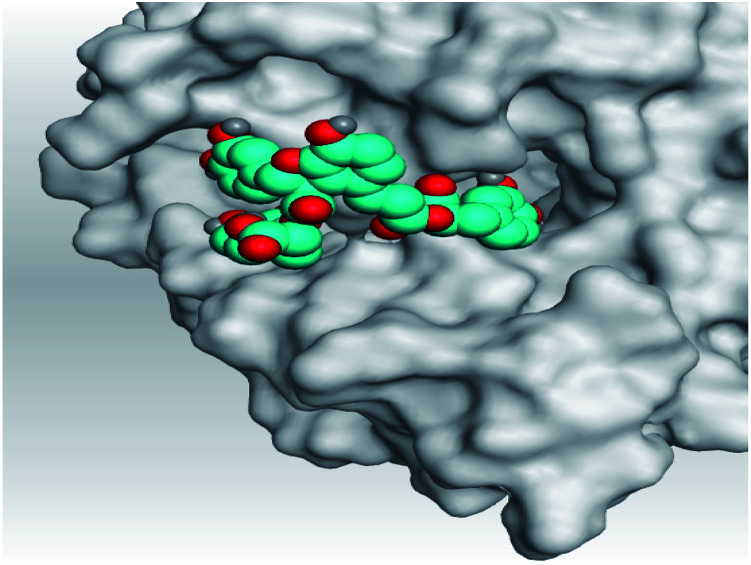
	Mpro	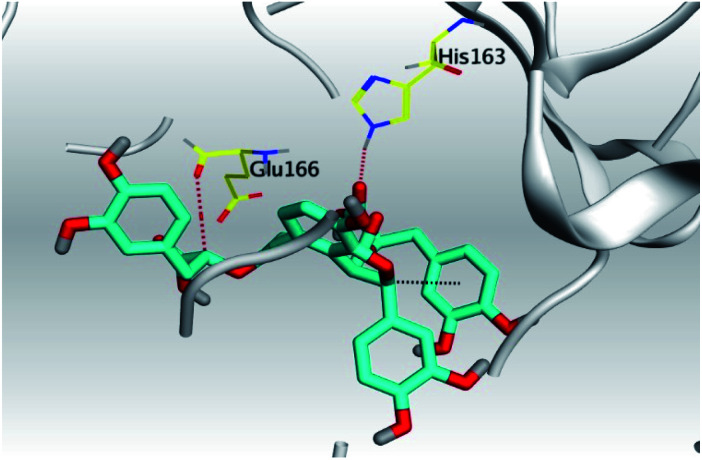	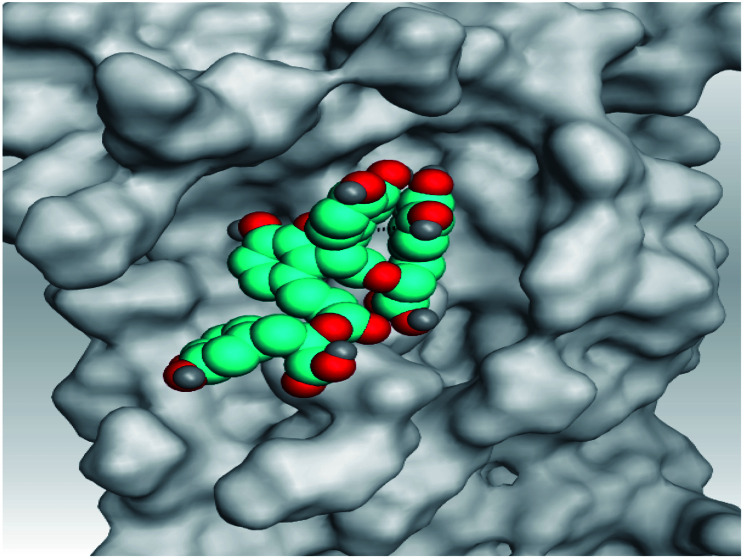
5	S	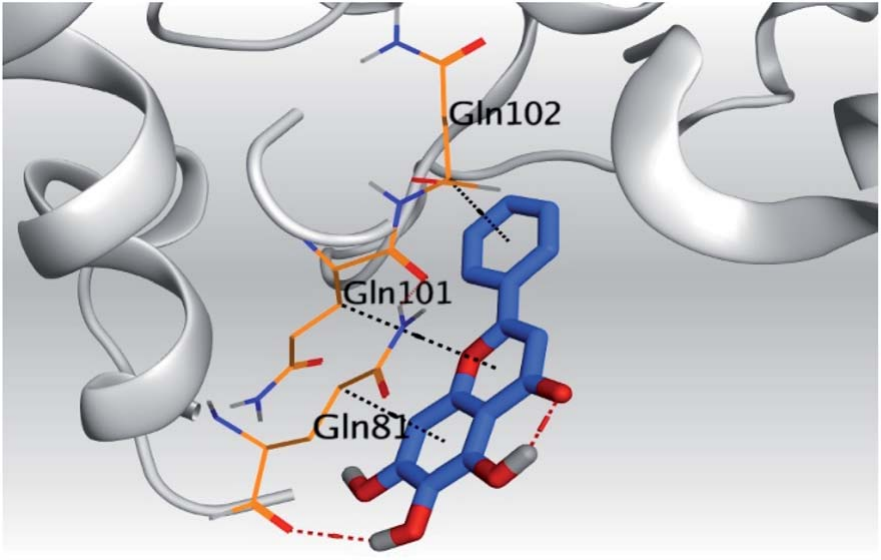	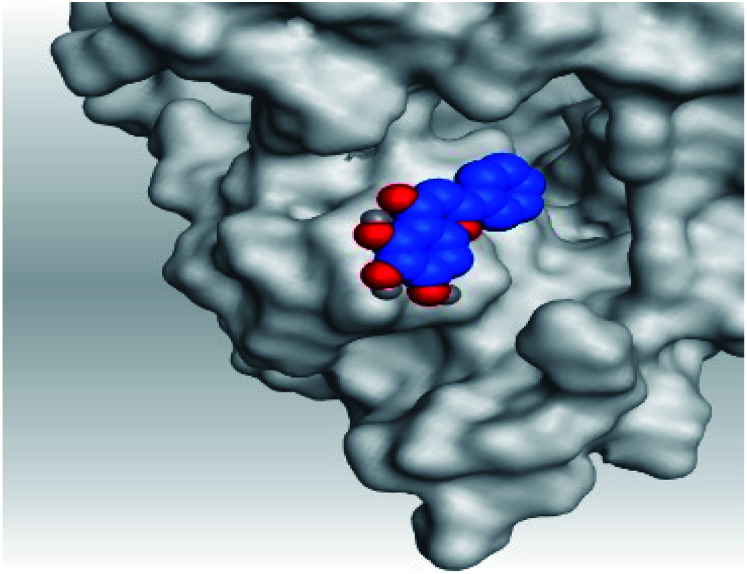
	Mpro	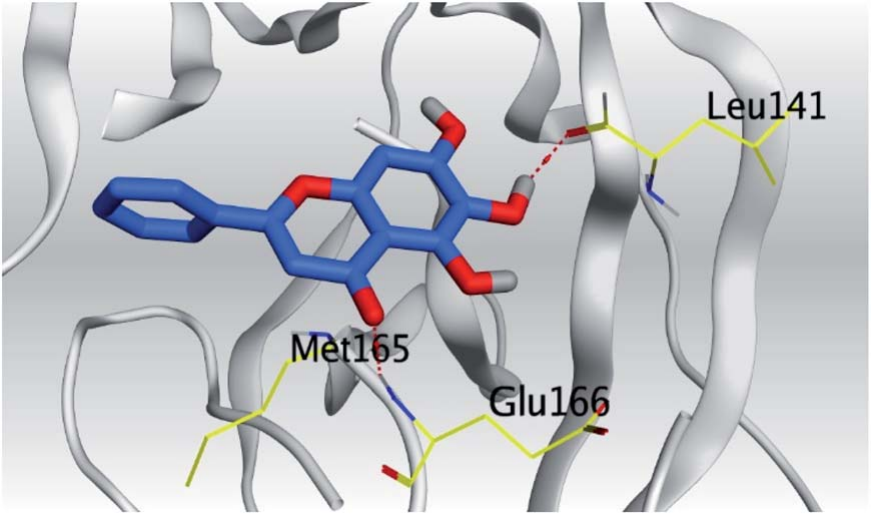	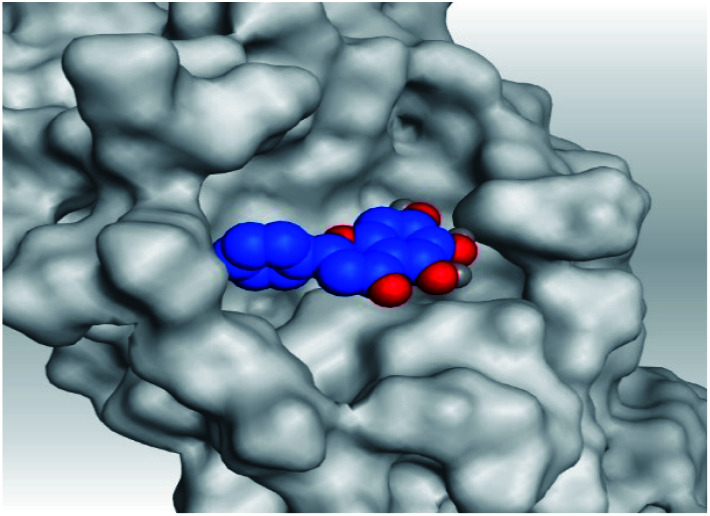
6	S	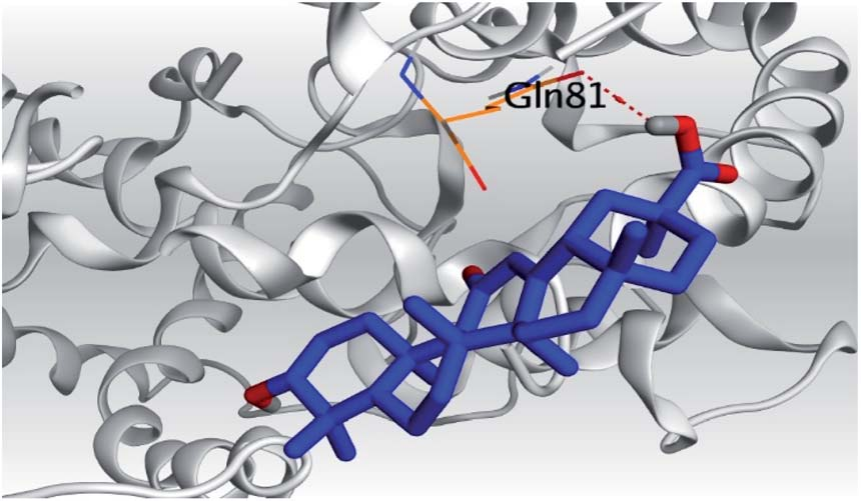	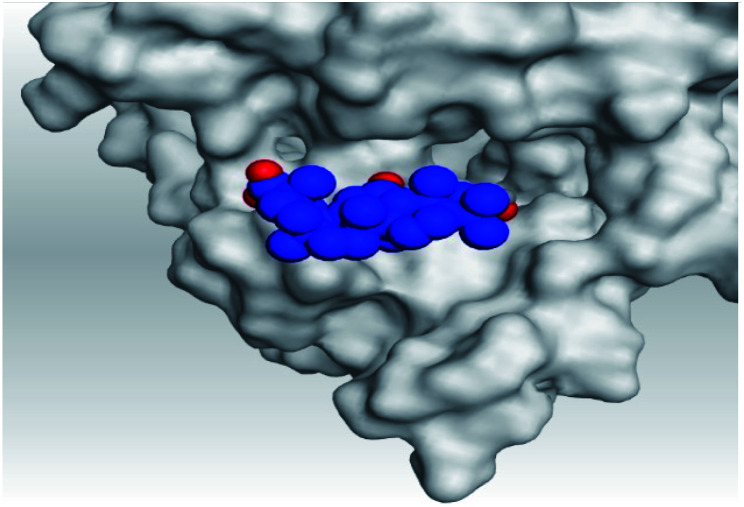
	Mpro	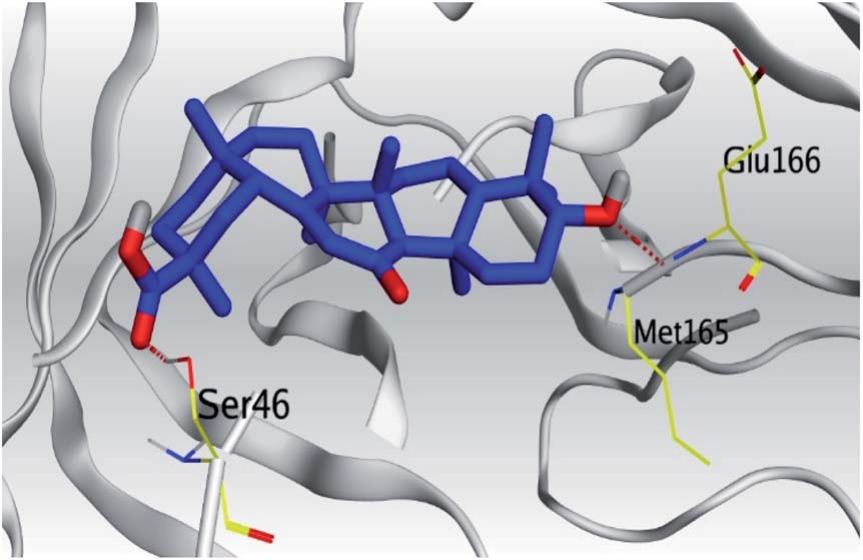	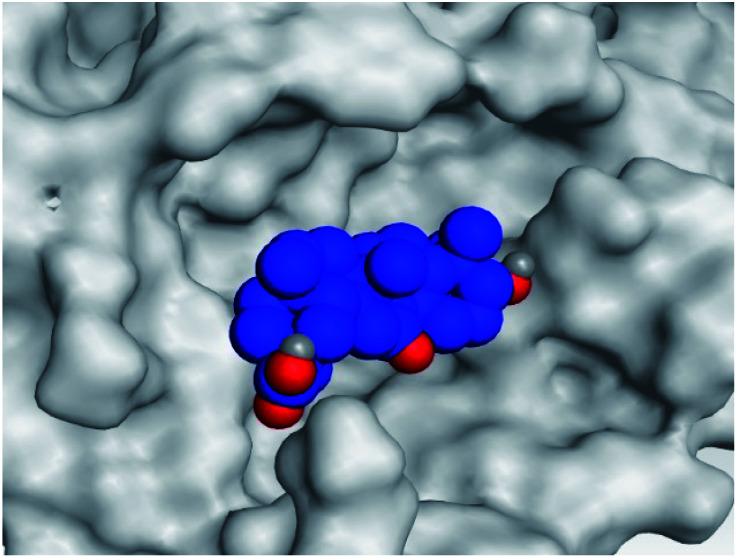
N3, 7	Mpro	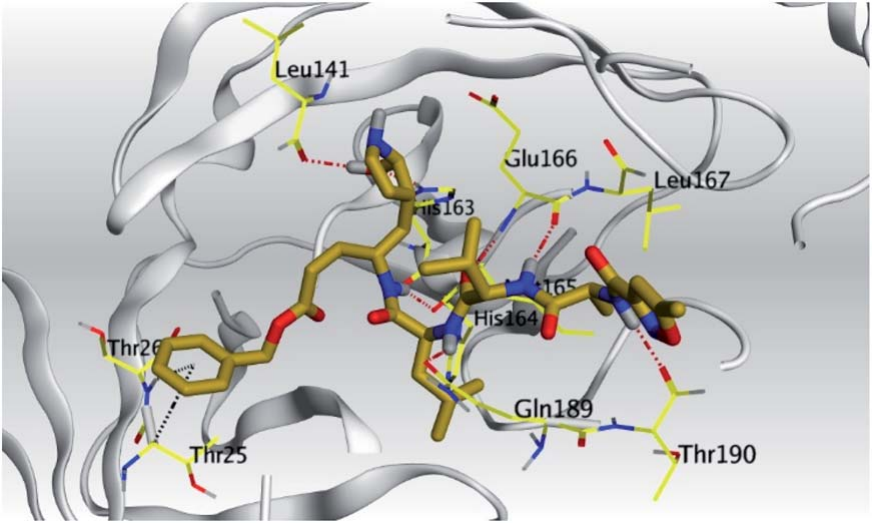	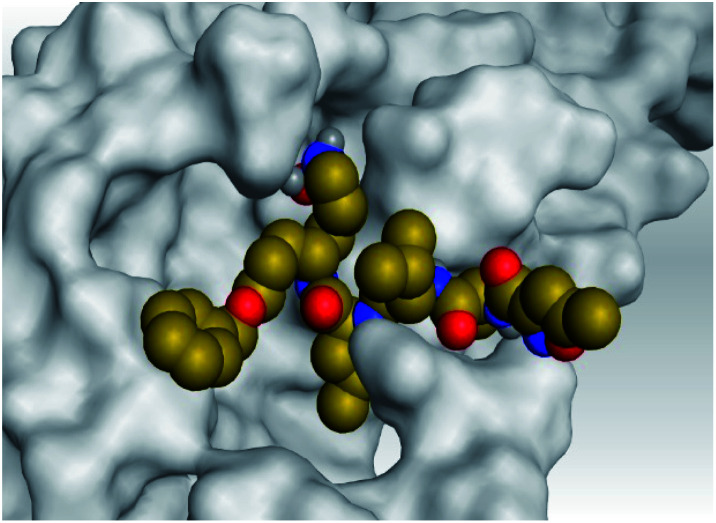

a H-bonds were represented by red dashed lines while H-pi bonds by black ones.

(a) Both salvianolic acid (4) and glycyrrhetinic acid (6) showed the best binding affinities towards the S and Mpro pockets of SARS-CoV-2 with scores equivalent to −7.85 and −9.23 kcal mol^−1^ for salvianolic acid, and −6.90 and −6.77 kcal mol^−1^ for glycyrrhetinic acid, respectively.

(b) Only tanshinone IIA (1) achieved two H-bond formations with Cys145 and His163 amino acids at 3.02 and 3.22 Å, respectively, (the two important amino acids forming the catalytic dyad of SARS-CoV-2 Mpro^49^) which indicating a greatly promising anti-SARS-CoV-2 intrinsic activity.

(c) Moreover, carnosic acid (2) and salvianolic acid (4) achieved one H-bond formation with Cys145 amino acid at 3.13 and 3.05 Å, respectively, (one of the two important amino acids for the catalytic dyad of SARS-CoV-2 Mpro^49^) which indicating predicted promising anti-SARS-CoV-2 intrinsic activities as well.

### 3.2. Molecular dynamics (MD) simulations

To confirm that these compounds are actually targeting the S and Mpro proteins and to inspect the stability of the docked compounds into the binding pockets of both the S and Mpro pockets of SARS-CoV-2, molecular dynamic simulations were performed. The salvianolic acid achieved the highest scores from the docking point of view; on the other hand, tanshinone IIA appeared to be the most active compound biologically; thus, salvianolic acid and tanshinone IIA complexes with both S protein (Sal–S and Tan–S) and Mpro protein (Sal–Mpro and Tan–Mpro) were subject to a 100 ns MD simulation. The co-crystallized N3 inhibitor of Mpro (N3–Mpro) was also subjected to a 100 ns simulation to be used as a reference in the case of Mpro stability and MM-GBSA energy calculations; unfortunately, the S protein has no co-crystallized ligand.

#### 3.2.1. RMSD analysis

The Root Mean Square Deviation (RMSD) is a quantitative measurement that describes the overall stability of the system during the simulation time by showing the deviation degree from the initial structure.

**Fig. 2:**
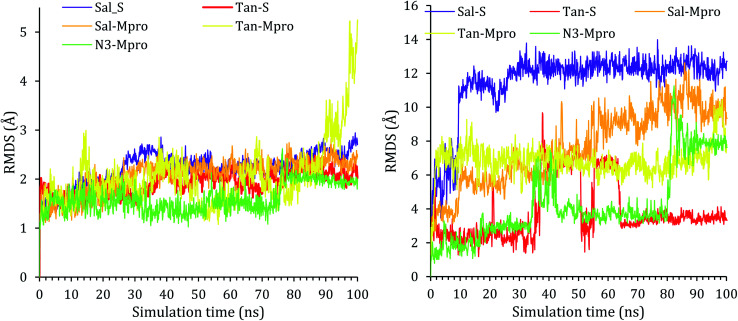
The RMSD of complex (left) and the ligands (right) as a function of simulation time.

The protein RMSD for all proteins showed early stability and reached a plateau at around 20 ns of the simulation time with RMSD less than 3 Å, and the only exception was for Tan–Mpro, which fluctuated at around 2.5 Å, at around 80 ns, the protein N-terminal start to completely flapped and change its orientation as it can be seen in Fig. ESI 1,† the same fluctuation was observed in N3–Mpro at around 80 ns of simulation time the N-terminal start to fluctuate and move around 0.6 Å, Fig. ESI 2.† All RMSDs of proteins are shown in Fig. 2.

The RMSD of the ligand was also reported with respect to their initial position in the active site of the protein and reported as a function of time in Fig. 2. A snapshot at 0 ns and 100 ns is reported in Fig. ESI 3–7.† As it can be seen from the ligands RMSD, salvianolic acid moved around 13 Å and 12 Å inside the S and Mpro, respectively. Salvianolic acid reaches equilibrium in the case of S protein, while it takes almost 60 ns to reach stability in the case of Mpro, due to the fact that salvianolic is quite a big molecule with MW of 716 and it has more than ten rotatable bonds, such a diffusion from the active site is still acceptable. In the case of tanshinone IIA, it showed more stability than salvianolic acid inside the S protein, with an RMSD of 3 Å, and reaches the plateau at an early stage, at around 35–70 ns tanshinone IIA tried to get deeper inside the active site which was not stable at the new position due to clashes, and losing of the Asn103, Ala193, and His195, as it will be clarified later. For the Tan–Mpro, it looks like the compound is affecting the conformation of the protein itself; as it will be discussed later, the tanshinone IIA starts to form new interactions with Arg188, which affect the structure of the protein; however, the Tan–Mpro showed high RMSD in overall.

Finally, the protein RMSD for the docked N3 inside its Mpro pocket of SARS-CoV-2 showed small initial fluctuations within the range of 1 Å from the start till reaching 70 ns of the simulation time. Then it showed a larger fluctuation accompanied with movement of the N3 by around 9 Å with respect to its initial position inside the active site.

#### 3.2.2. RMSF analysis

The Root Mean Square Fluctuation (RMSF) is useful to get more deep insights regarding the flexibility observed in the residues of the receptor protein in the presence of its proposed inhibitor molecule. It clarifies the local changes within the protein structure throughout the simulation time.

**Fig. 3:**
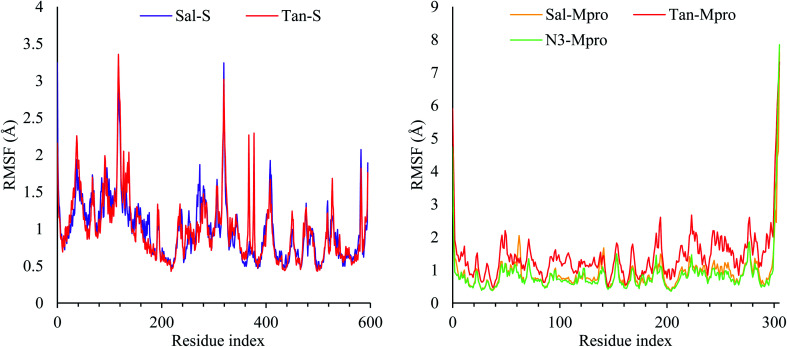
The RMSF of the S protein (left) and the Mpro protein (right).

Due to the fact the S protein structure is more rigid, it showed high stability with RMSF less than 3.5 Å. Three notable fluctuations were noted at residues 130–140 and 330–340, which, as expected, was a loop with no rigid conformation. In the case of Mpro, most of the protein was stable during simulation except for the N- and C-terminal, which fluctuates up to 8 Å. The RMSFs of the five complexes were reported in Fig. 3.

#### 3.2.3. Binding interactions histogram and heat map analysis

**Fig. 4:**
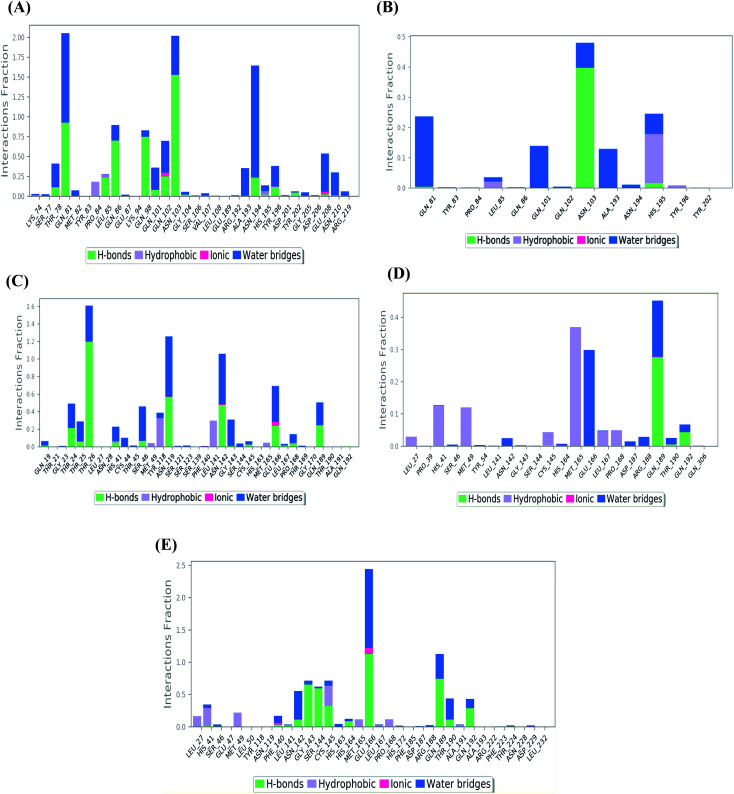
Histogram describing the binding interactions between the protein and its ligand during the simulation time of 100 ns for (A) Sal–S and (B) Tan–S, (C) Sal–Mpro, (D) Tan–Mpro, and (E) N3–Mpro.

The binding interactions histogram for each studied protein–ligand complex during the simulation time of 100 ns has been depicted in Fig. 4.

In the case of Sal–S, the amino acids Gln81, Gln86, Gln98, and Asn103 contributed mainly to the hydrogen bonding interactions, almost 70% of simulation time, with Gln81 and Asn103 being able to form more than one hydrogen bond during simulation; however, Gln81 and Asn194 contributed mainly *via* water bridges hydrogen bonds to the docked Sal. On the other side, Pro84, Leu85, and His195 were able to contribute hydrophobically. Moreover, the ionic interactions were represented only through Gln102, Asn194, and Asp206 (Fig. 4A). On the other hand, Tan–S binding interactions showed that the hydrogen bonding was represented by only Asn103 and His195. Hydrophobic interactions to Tan were through Leu85, His195 (∼35%), and Tyr196 amino acids only. Also, Gln81, Gln101, and Ala193 formed the most water bridges hydrogen bonds (∼20%), and no ionic interactions were observed 116 for the docked Tan inside the S pocket of SARS-CoV-2 (Fig. 4B). It was obvious that Asn103 amino acid was interacting the most with Sal and Tan inside the binding pocket of SARS-CoV-2 S protein.

Analyzing the binding interactions in the case of Sal–Mpro, it was clear that Thr26, Asn119, and Asn142 were responsible for most of the hydrogen bonding interactions, with Thr26 interacting more than 160% of the time through more than one hydrogen bond. Hydrophobic interactions were only through Tyr118 and Leu141 amino acids (∼20%), and ionic interactions were only with Asn142 and Glu166 amino acids. Also, Thr26, Asn119, Asn142, and Glu166 were the main amino acids contributing to the water bridges hydrogen bond (Fig. 4C). Furthermore, the Tan–Mpro complex showed hydrogen bonding interactions with Gln189 (∼50%) and Gln192 (∼10%) amino acids only. Their hydrophobic interactions were mainly through His41, Met49, and Met165 (∼40%), and their water bridges hydrogen bonds were mainly represented by Glu166 and Gln189 amino acids (∼38%) (Fig. 4D). On the other hand, N3–Mpro as a reference showed hydrogen bonding interactions with Gly143 (∼80%), Ser144 (∼75%), Glu166 (∼250%), and Gln189 (∼110%) as the main contributing amino acids. Also, His41 and Cys145 contributed mainly to the hydrophobic interactions, and Phe140, Asn142, and Glu166 contributed only to the ionic interactions to the N3 pose. However, Glu166 showed the main water bridges hydrogen bond interactions (Fig. 4E).

**Fig. 5:**
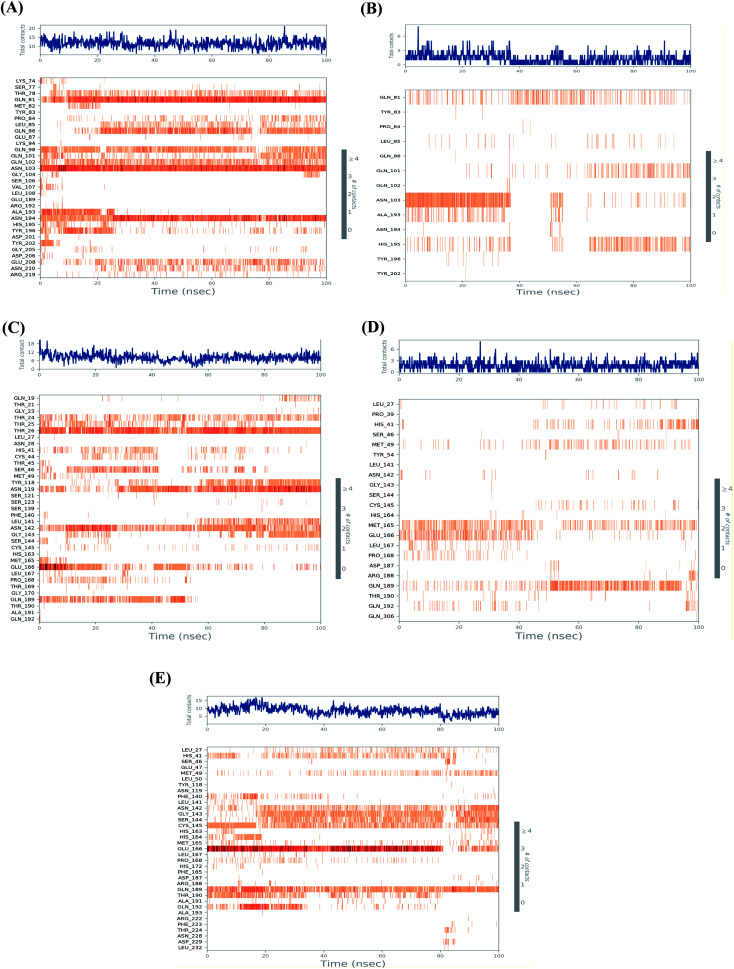
Heat map representing the total number of protein–ligand contacts during the simulation time of 100 ns for (A) Sal–S and (B) Tan–S, (C) Sal–Mpro, (D) Tan–Mpro, and (E) N3–Mpro.

Fig. 5 shows the heat map for the total number of contacts and interactions of salvianolic acid and tanshinone IIA within the S and Mpro pockets, besides that of the N3 inhibitor inside the Mpro pocket of SARS-CoV-2 protein as a reference. It was observed that the main binding for salvianolic acid inside the S pocket was through Asn103, Gln81, and Asn194 (Fig. 5A). Whatever, the main binding residue for tanshinone IIA inside the same pocket was found to be Asn103 throughout (35–40%) of the simulation time (Fig. 5B). This indicates the great importance of Asn103 amino acid inside the binding pocket of SARS-CoV-2 S protein in the interactions with its proposed inhibitors.

On the other hand, the main binding amino acids for salvianolic acid inside its Mpro binding pocket were found to be Thr26, Asn119, and Asn142 during (>70%) of the simulation time (Fig. 5C). However, the higher number of contacts for tanshinone IIA within the Mpro binding pocket was observed with Met165, Glu166, and Gln189 (>25%) throughout the simulation period (Fig. 5D).

Finally, the co-crystallized N3 inhibitor inside the Mpro binding pocket of SARS-CoV-2 showed greater interactions with Glu166, Gln189, Cys145, Gly143, and Ser144 amino acids (>65%) (Fig. 5E). Again, Glu166 amino acid was observed to be of great importance towards the interactions of SARS-CoV-2 Mpro receptor to its proposed inhibitors.

#### 3.2.4. Ligand properties study analysis

**Fig. 6:**
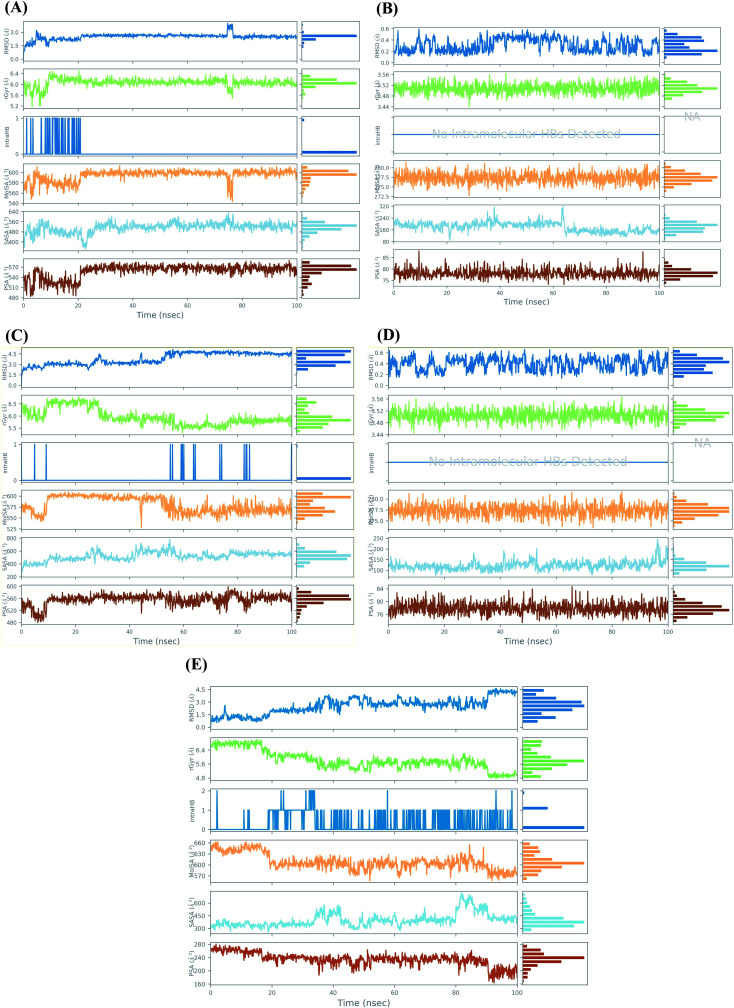
Ligand properties study during the simulation time of 100 ns for (A) Sal–S and (B) Tan–S, (C) Sal–Mpro, (D) Tan–Mpro, and (E) N3–Mpro.

Ligand properties study describes the ligand Root Mean Square Deviation (RMSD), radius of Gyration (rGyr), intramolecular Hydrogen Bonds (intraHB), Molecular Surface Area (MolSA), Solvent Accessible Surface Area (SASA), and Polar Surface Area (PSA) as depicted in Fig. 6.

For the docked pose of Sal–S, the RMSD was within the range of 2 Å. Its rGyr-which measures the extendedness of a ligand-was in the range of (5.2–6.4 Å), and the equilibrium was around 6 Å. Also, its intraHB-representing the number of internal hydrogen bonds (HB) within a ligand molecule-was observed from the start of the simulation until 20 ns. The MolSA-which is equivalent to a van der Waals surface area calculated with a 1.4 Å probe radius-showed fluctuations from the start of the simulation till reaching its equilibrium at 20 ns, and its range was observed in between (540–600 Å^2^) with an equilibrium around 600 Å^2^. Moreover, the surface area of Sal accessible by a water molecule (SASA) showed heavy fluctuations up to 25 ns, showed equilibrium till the end of the simulation time. The SASA range was between 320 to 640 Å^2^, and the equilibrium was around 520 Å^2^. Furthermore, the PSA, which refers to the SASA in salvianolic acid, is contributed only by oxygen and nitrogen atoms. Its range was around 480–590 Å^2^, and the equilibrium was around 530 Å^2^ (Fig. 6A). On the other hand, concerning the docked pose of Tan–S, the RMSD was within the range of 4 Å. Its rGyr was in the range of (3.44–3.56 Å), indicating high compactness of the protein structure, and the equilibrium was around 3.50 Å throughout the simulation time. Also, no intraHB was observed all over the simulation. The MolSA showed low fluctuations throughout the simulation time, and its range was observed in between (272.5–282 Å^2^) with an equilibrium around 278.5 Å^2^. Moreover, the SASA of tanshinone IIA showed moderate fluctuations throughout the simulation time, its range was between 80 to 320 Å^2^, and the equilibrium was around 200 Å^2^. Furthermore, its PSA range was around 70–90 Å^2^, and the equilibrium was around 78 Å^2^ (Fig. 6B).

However, analyzing the docked pose of Sal–Mpro, its RMSD was within the range of 3 Å. Its rGyr was in the range of (5–7 Å), and the equilibrium was around 5.8 Å at the end of the simulation time (>60 ns). Also, its intraHB was observed more at the second half of the simulation (>50 ns). The MolSA showed initial fluctuations from the start of the simulation till reaching its equilibrium at 10 ns and was returned to fluctuations again at 50 ns till the end of the simulation time. Its range was observed in between (525–620 Å^2^) with an equilibrium around 600 Å^2^. Moreover, the SASA of salvianolic acid showed moderate fluctuations throughout the simulation time, its range was between 300 to 800 Å^2^, and the equilibrium was around 500 Å^2^. Furthermore, its PSA range was around 480–600 Å^2^, and the equilibrium was around 560 Å^2^ (Fig. 6C). Furthermore, the RMSD of the docked pose (Tan–Mpro) was within the range of 4 Å. Its rGyr was in the range of (3.44–3.56 Å), indicating high compactness of the protein structure, and the equilibrium was around 3.51 Å throughout the simulation time. Also, no intraHB was observed all over the simulation. The MolSA showed low fluctuations throughout the simulation time, and its range was observed in between (272.5–282 Å^2^) with an equilibrium around 277.5 Å^2^. Moreover, the SASA of tanshinone IIA showed moderate fluctuations throughout the simulation time, its range was between 60 to 250 Å^2^, and the equilibrium was around 125 Å^2^. Furthermore, its PSA range was around 74–84 Å^2^, and the equilibrium was around 78 Å^2^ (Fig. 6D). It is worth mentioning that the ligand properties study for tanshinone IIA inside both the S and Mpro pockets of SARS-CoV-2 showed nearly the same results, which appeared to be identical in most cases, indicating similar behavior of tanshinone IIA throughout the simulation time in both cases.

Finally, the docked pose of N3–Mpro as a reference showed an RMSD within the range of 3.5 Å. Its rGyr was in the range of (4.8–7 Å), and the equilibrium was around 5.8 Å at the start (<20 ns) and the end of the simulation time (>60 ns). The intraHB was distributed throughout the simulation time, being more obvious after exceeding the first 20 ns. The MolSA showed initial fluctuations from the start of the simulation till reaching its equilibrium at 20 ns and was returned to fluctuations again at 85 ns till the end of the simulation time. Its range was observed in between (550–660 Å^2^) with an equilibrium around 610 Å^2^. The SASA of N3 showed higher fluctuations at the end of the simulation time (>80 ns), its range was between 250 to 750 Å^2^, and the equilibrium was around 375 Å^2^. Also, its PSA range was around 160–280 Å^2^, and the equilibrium was around 240 Å^2^ (Fig. 6E).

### 3.3. MD trajectory analysis and prime MM-GBSA calculations

The average MM-GBSA binding energy was applied to calculate Coulomb, covalent binding, hydrogen-bonding, lipophilic, generalized Born electrostatic solvation, and van der Waals energies through applying the thermal_mmgbsa.py python script of Schrödinger. All the obtained results are described in Table 3.

**Table 3:** Prime MM-GBSA energies for Sal and Tan binding at both active sites of SARS-CoV-2 (S and Mpro) and N3 inhibitor of Mpro^a^

Complex	Δ*G* Binding	Coulomb	Covalent	H-bond	Lipo	Bind packing	Solv_GB	vdW	St. dev.
Sal–S	−49.43	112.93	3.52	−4.16	−10.45	−2.53	−108.77	−39.97	7.20
Tan–S	−42.61	−1.80	0.75	−0.18	−13.03	−3.53	11.49	−36.32	3.08
Sal–Mpro	−45.75	5.95	3.77	−2.04	−15.88	−1.36	2.09	−38.28	5.52
Tan–Mpro	−43.58	−4.93	0.77	−0.25	−12.71	−2.30	11.35	−35.51	4.87
N3–Mpro	−53.29	−23.32	3.22	−2.06	−11.38	−0.72	27.05	−46.09	9.46

a Coulomb: Coulomb energy; covalent: covalent binding energy; H-bond: hydrogen-bonding energy; lipo: lipophilic energy; solv_GB: generalized born electrostatic solvation energy; vdW: van der Waals energy; St. dev.: standard deviation.

### 3.4. *In vitro* results

**Fig. 7:**
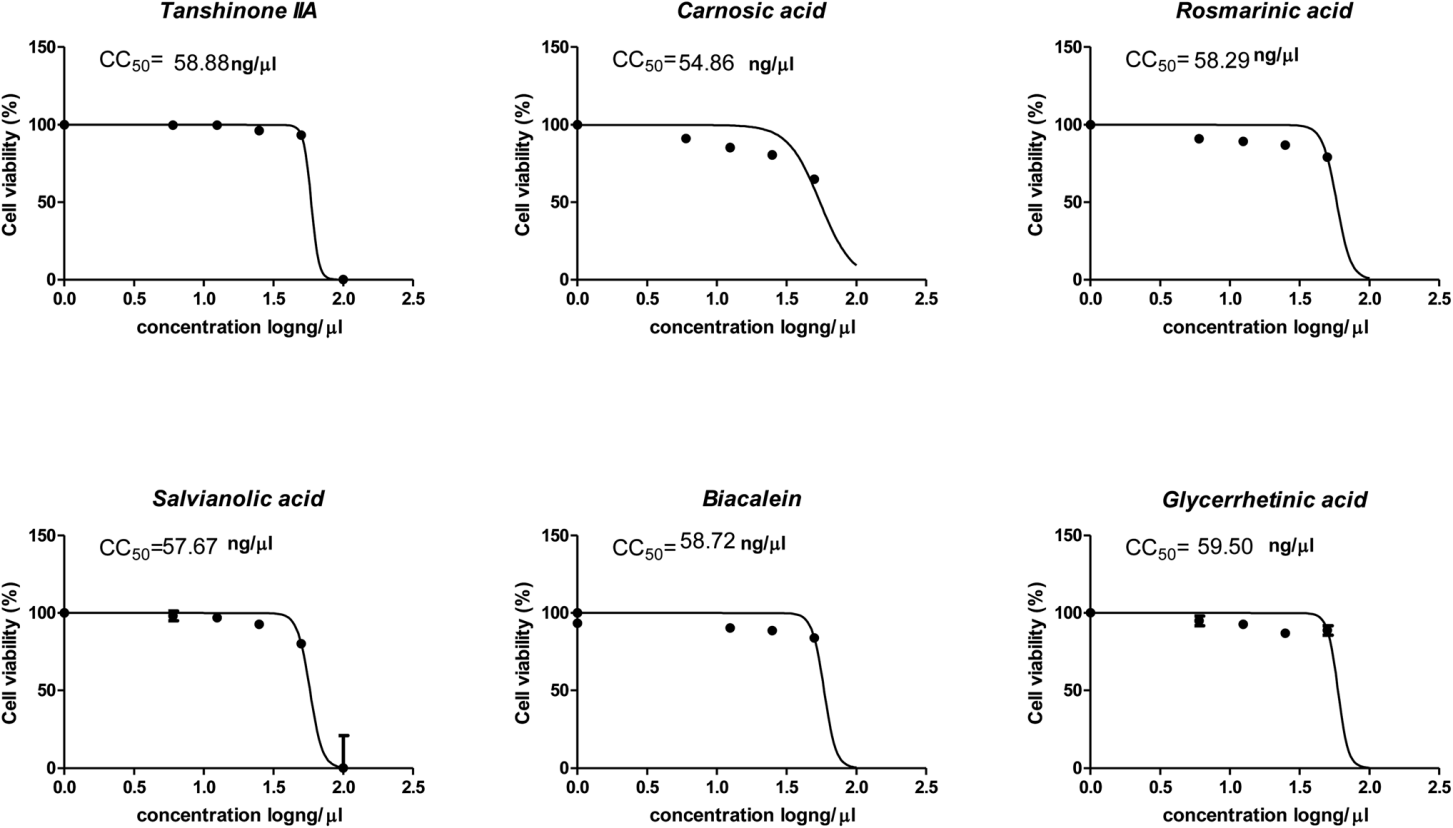
Graph of cytotoxicity concentration 50 (CC_50_) on Vero E6 cells using nonlinear regression analysis of GraphPad Prism software (version 5.01) by plotting log cell viability *versus* normalized response (variable slope).

**Fig. 8:**
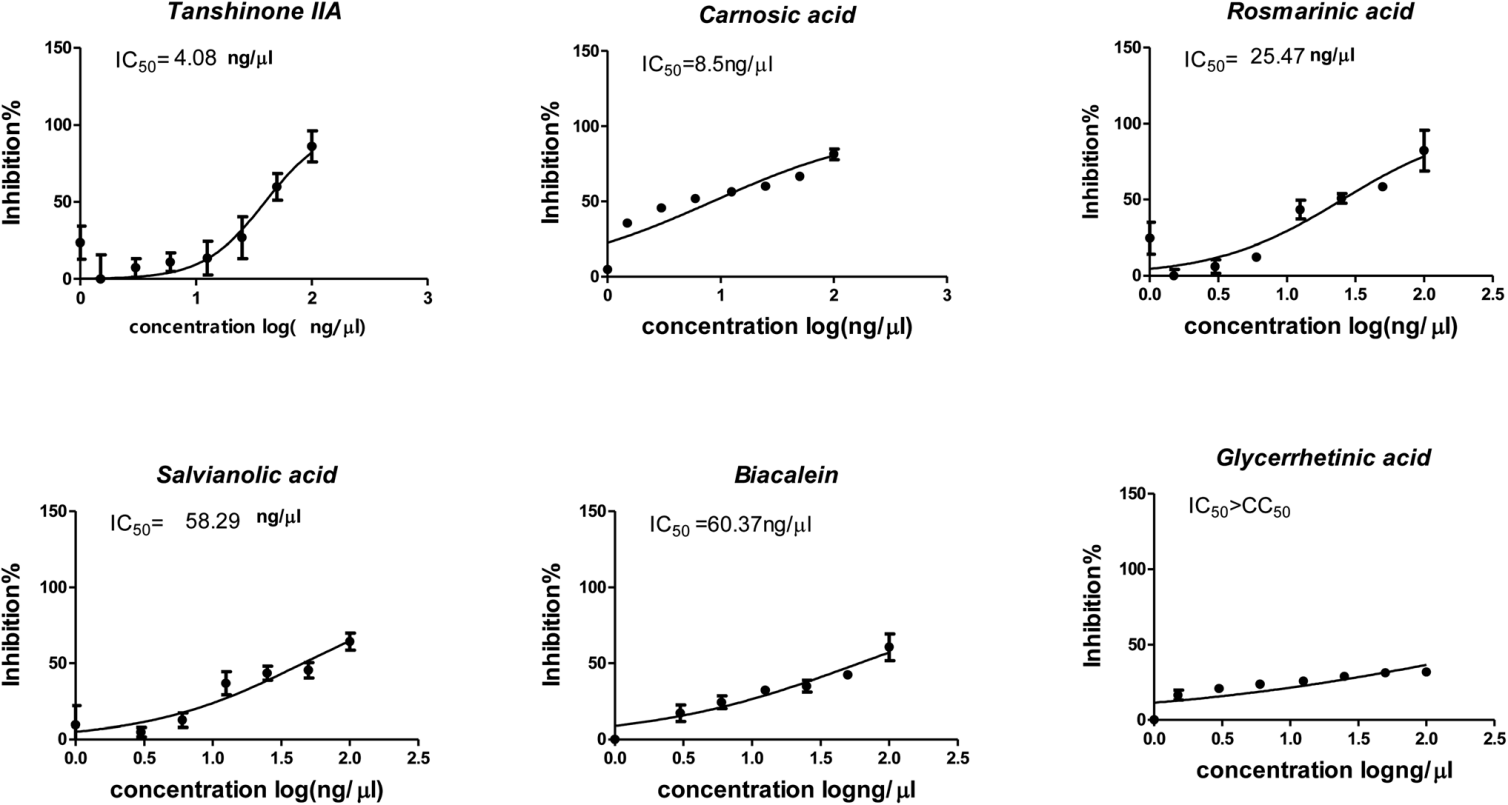
Graph of inhibitory concentration 50 (IC_50_): Antiviral activity against Severe Acute Respiratory Syndrome Coronavirus 2 (SARS-CoV-2) (hCoV-19/Egypt/NRC-03/2020, accession number on GSAID: EPI_ISL_430820) Vero E6 cells using nonlinear regression analysis of GraphPad Prism software (version 5.01) by plotting log inhibitory *versus* normalized response (variable slope).

The cytotoxicity CC_50_ of the tested compounds (1–6) on Vero E6 cells (Fig. 7) showed that the safety concentrations for each compound on the cells to be used in other tests. Inhibitory concentration (IC_50_) (Fig. 8) to calculate the dose that causes inhibition to 50% pathogenicity of the virus. The best one that achieved the greatly promising anti-SARS-CoV-2 activity was tanshinone IIA (1) with IC_50_ equal 4.08 ng μl^−1^ and the second one was carnosic acid (2) showed promising IC_50_ values equal 8.5 ng ml^−1^. The compound that had a medium effect was rosmarinic acid (3) with IC_50_ equals 25.47 ng μl^−1^. Salvianolic acid (4) and baicalein (5) showed low activity against-SARS-CoV-2 with IC_50_ values equal 58.29 ng μl^−1^ and 60.2 ng μl^−1^, respectively. On the other hand, and Glycerrhetinic acid (6) not showed apparent effects against SARS-CoV-2 while IC_50_ > CC_50_. Plaque reduction assay (Tables ESI 1 and ESI 2†) with pictures of plates confirmed the results that tanshinone IIA (1) is the best one that has antiviral activity against SARS-CoV-2 and also carnosic acid (2) has more than 90% of inhibition for virus propagation.

**Fig. 9:**
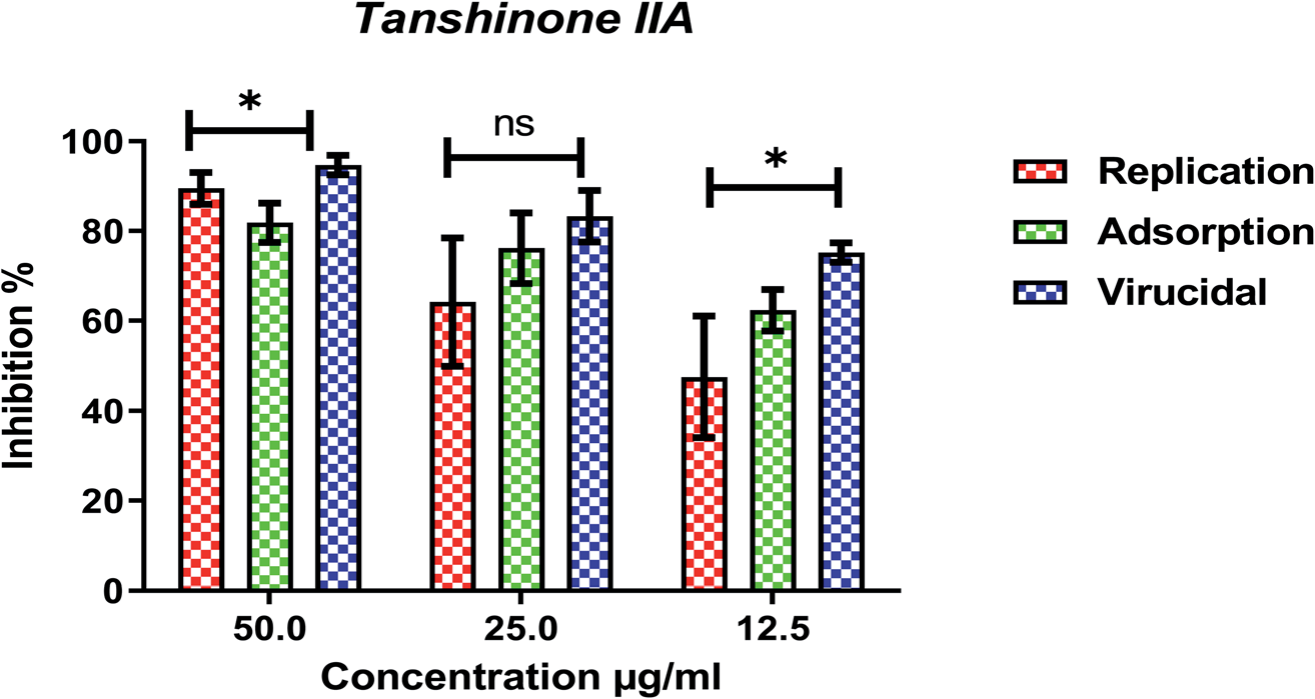
Mode of action for tanshinone IIA against SARS-CoV-2. The significant differences are indicated (* = *p* < 0.05, and non-significant = ns).

**Fig. 10:**
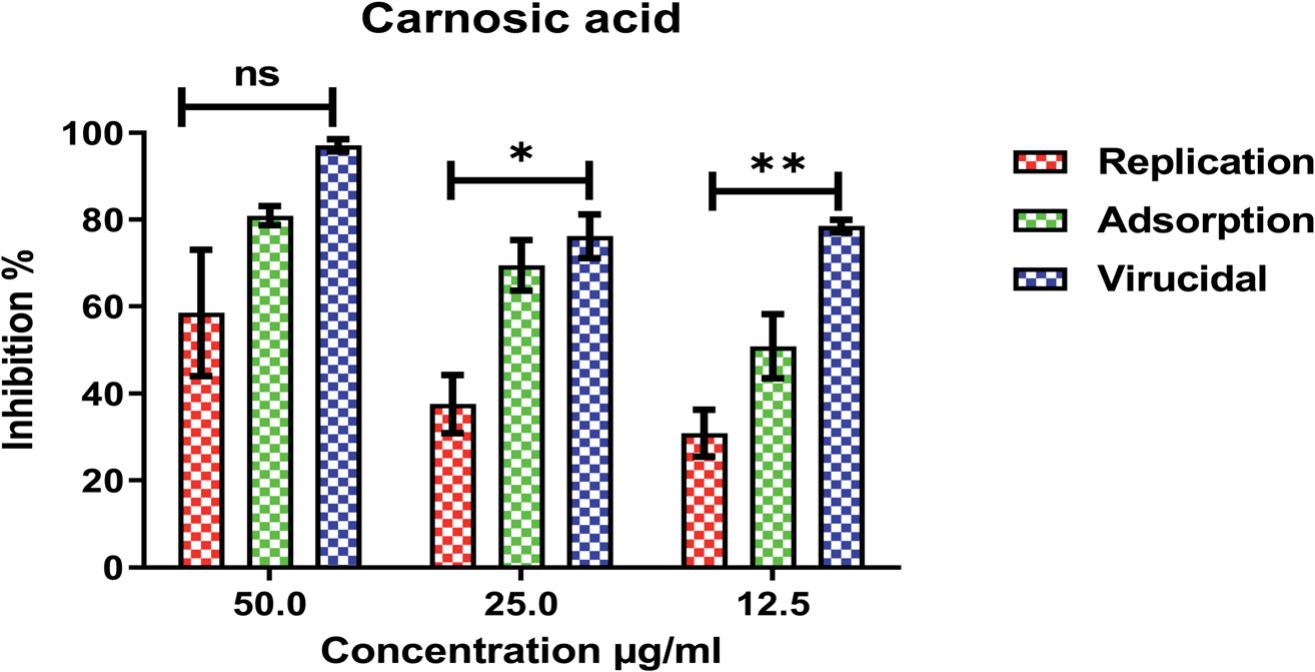
Mode of action for carnosic acid against SARS-CoV-2. The significant differences are indicated (* = *p* < 0.05, ** = *p* < 0.01, and non-significant = ns).

**Fig. 11:**
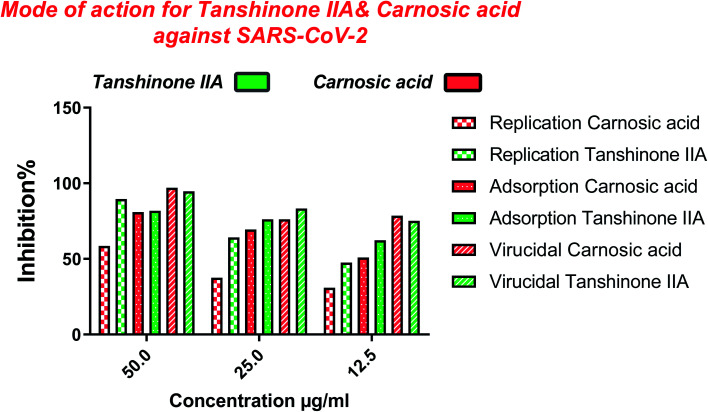
Graphical representation describing the proposed modes of action for both tanshinone IIA and carnosic acid against SARS-CoV-2.

Furthermore, to know the mechanism of action for the tested compounds towards SARS-CoV-2, it was necessary to examine the mode of action for the most two promising compounds tanshinone IIA and carnosic acid (Fig. 9 and 10). Interestingly, tanshinone IIA and carnosic acid had a combination of viral inhibitory effects on the tested SARS-CoV-2 at different viral stages. Both compounds (1 and 2) showed significant virucidal activity at concentration 12.5 μg (*p* < 0.05). Tanshinone IIA had a 94% virucidal effect against SARS-CoV-2 at a concentration of 50 μg and about 89% and 81% for virus replication and adsorption stage, respectively. No significant differences (*p* > 0.05) were observed among the three tested modes of action against SARS-CoV-2 at concentration 25 μg of tanshinone IIA. Although no significant differences (*p* > 0.05) were observed among the three tested modes of actions against SARS-CoV-2 at concentration 50 μg of carnosic acid, it exhibited the virucidal effect with more than a 97% viral inhibitory effect and an approximately 88% inhibitory effect on virus adsorption as well as 58% inhibitory effect on virus replication. This recommended the predicted activity of the two compounds against the S protein of SARS-CoV-2 rather than its Mpro protein. A graphical representation describing the proposed modes of action for both tanshinone IIA and carnosic acid against SARS-CoV-2 is depicted in Fig. 11.

## 4. Conclusion

Molecular docking studies recommended the better affinities of both salvianolic acid B (4) and glycyrrhetinic acid (6) between the tested six compounds against the S and Mpro receptor pockets of SARS-CoV-2 as well. On the other hand, the performed *in vitro* tests confirmed the superior activity of tanshinone IIA (1) with IC_50_ equals 4.08 ng μl^−1^ which was confirmed by its 100% inhibition in the plaque reduction assay at the four applied concentrations. So, salvianolic acid achieved the highest scores from the docking point of view and tanshinone IIA appeared to be the most active compound biologically. Therefore, salvianolic acid and tanshinone IIA complexes with both S protein (Sal–S and Tan–S) and Mpro protein (Sal–Mpro and Tan–Mpro) were subject to a 100 ns MD simulation which confirmed the docking results and gave deep insights into their binding behaviors as well. Also, both carnosic acid (2) and rosmarinic acid (3) showed promising IC_50_ values of 15.37 and 25.47 ng μl^−1^ and achieved 92.5% and 70.2% inhibition in the plaque reduction assay with the highest concentrations, respectively. However, salvianolic acid (4) showed a weak anti-SARS-CoV-2 activity with an IC_50_ value of 58.29 ng μl^−1^. Moreover, the mode of action for the most two promising compounds, tanshinone IIA (1) and carnosic acid (2), to understand the mechanism of their antiviral activity towards SARS-CoV-2 showed a very promising virucidal activity for both compounds with a most prominent inhibitory effect on the viral adsorption rather than its replication. This clarifies the predicted activity of the two compounds against the S protein of SARS-CoV-2 rather than its Mpro protein. Our findings could put a new spot to rearrange these compounds based on their actual *in vitro* activities against SARS-CoV-2 and to search for the reasons behind the great differences between their *in silico* and *in vitro* results against SARS-CoV-2. Finally, we recommend further advanced preclinical and clinical studies especially for tanshinone IIA (1) to be rapidly applied in COVID-19 management either alone or in combination with carnosic acid (2), rosmarinic acid (3), and/or salvianolic acid (4).

## Author contributions

Conceptualization: Dalia Elebeedy and Ahmed A. Al-karmalawy; data curation: Ahmed Kandeil, Aml Ghanem, Omnia Kutkat, Marwa A. Saleh, and Ahmed A. Al-karmalawy; formal analysis: Ahmed Kandeil, Aml Ghanem, and Omnia Kutkat; funding acquisition: Dalia Elebeedy and Ingy Badawy; investigation: Dalia Elebeedy and Ahmed A. Al-karmalawy; methodology: Ahmed Kandeil, Omnia Kutkat, Radwan Alnajjar, Marwa A. Saleh, and Ahmed A. Al-karmalawy; project administration: Dalia Elebeedy and Ahmed A. Al-karmalawy; resources: Dalia Elebeedy and Ahmed I. Abd El Maksoud; software: Radwan Alnajjar, and Ahmed A. Al-karmalawy; supervision: Dalia Elebeedy, Ingy Badawy, and Ahmed A. Al-karmalawy; validation: Ahmed Kandeil and Ahmed A. Al-karmalawy; visualization: Ahmed Kandeil, Radwan Alnajjar, and Ahmed A. Al-karmalawy; writing – original draft: Dalia Elebeedy, Walid Elkhatib, Aml Ghanem, Omnia Kutkat, and Ahmed A. Al-karmalawy; writing – review & editing: Dalia Elebeedy and Ahmed A. Al-karmalawy. All authors approved the final version of the manuscript.

## Conflicts of interest

The authors declare no conflict of interest.

## Supplementary Material

RA-011-D1RA05268C-s001
