# Supplementary material for: Anti-SARS-CoV-2 activities of tanshinone IIA, carnosic acid, rosmarinic acid, salvianolic acid, baicalein, and glycyrrhetinic acid between computational and in vitro insights
Source: RSC Adv. 2021 Sep 1;11(47):29267–86. doi: 10.1039/d1ra05268c (PMC9040650; doi:10.1039/d1ra05268c)
Supplement: RA-011-D1RA05268C-s001 [file RA-011-D1RA05268C-s001.pdf]

Electronic Supplementary Material (ESI) for RSC Advances.
This journal is © The Royal Society of Chemistry 2021

Anti-SARS-CoV-2 Activities of Tanshinone IIA, Carnosic acid, Rosmarinic acid, Salvianolic acid, Baicalein, and Glycyrrhetinic acid between Computational and *In Vitro* Insights

Dalia Elebeedy[a], Walid F. Elkhatib[b,c], Ahmed Kandeil[d], Aml Ghanem[e], Omnia Kutkat[d], Radwan Alnajjar[f,g], Marwa A. Saleh[h], Ahmed I. Abd El Maksoud[e], Ingy Badawy[a], and Ahmed A. Al-karmalawy[i]*

[a] College of Biotechnology, Misr University for Science and Technology (MUST), 6th of October City, Egypt.

[b] Department of Microbiology & Immunology, Faculty of Pharmacy, Galala University, New Galala city, Suez, Egypt.

[c] Microbiology and Immunology Department, Faculty of Pharmacy, Ain Shams University, African Union Organization St., Abbassia, Cairo 11566, Egypt.

[d] Center of Scientific Excellence for Influenza Viruses, National Research Centre, Giza 12622, Egypt.

[e] Department of Molecular Biology, Genetic Engineering and Biotechnology Research Institute, University of Sadat City, Sadat City, Egypt.

[f] Department of Chemistry, Faculty of Science, University of Benghazi, Benghazi, Libya.

[g] Department of Chemistry, University of Cape Town, Rondebosch 7701, South Africa.

[h] Department of Pharmaceutical Organic Chemistry, Faculty of Pharmacy (Girls), Al-Azhar University, Nasr City, Cairo, Egypt.

[i] Department of Pharmaceutical Medicinal Chemistry, Faculty of Pharmacy, Horus University-Egypt, New Damietta 34518, Egypt.

*Corresponding author:

Ahmed A. Al-Karmalawy: Email: akarmalawy@horus.edu.eg

ORCID: 0000-0002-8173-6073

Materials and Methods

SI 1: Molecular dynamics simulations

The MD simulations were carried out using Desmond simulation package of Schrödinger LLC.[1] The NPT ensemble with the temperature 300 K and a pressure 1 bar was applied in all runs. The simulation length was 100 ns with a relaxation time 1 ps for the ligands. The OPLS3 force field parameters were used in all simulations.[2] The cutoff radius in Coulomb interactions was 9.0 Å. The orthorhombic periodic box boundaries were set 10 Å away from the protein atoms. The water molecules were explicitly described using the transferable intermolecular potential with three points (TIP3P) model.[3, 4] Salt concentration set to 0.15 M NaCl and was built using the System Builder utility of Desmond.[5] The Martyna−Tuckerman−Klein chain coupling scheme with a coupling constant of 2.0 ps was used for the pressure control and the Nosé−Hoover chain coupling scheme for the temperature control.[6, 7] Nonbonded forces were calculated using a RESPA integrator where the short-range forces were updated every step and the long-range forces were updated every three steps. The trajectories were saved at 20 ns intervals for analysis. The behavior and interactions between the ligands and protein were analyzed using the Simulation Interaction Diagram tool implemented in Desmond MD package. The stability of MD simulations was monitored by looking on the RMSD of the ligand and protein atom positions in time.

SI 2: MD trajectory analysis and prime MM-GBSA calculations

Simulation interactions diagram panel of Maestro software was used to monitoring interactions contribution in the ligand-protein stability. The molecular mechanics generalized born/solvent accessibility (MM – GBSA) was performed to calculate the ligand binding free energies and ligand strain energies for docked compounds over the last 25 ns with thermal_mmgbsa.py python script provided by Schrodinger which takes a Desmond trajectory file, splits it into individual snapshots, runs the MM-GBSA calculations on each frame, and outputs the average computed binding energy.

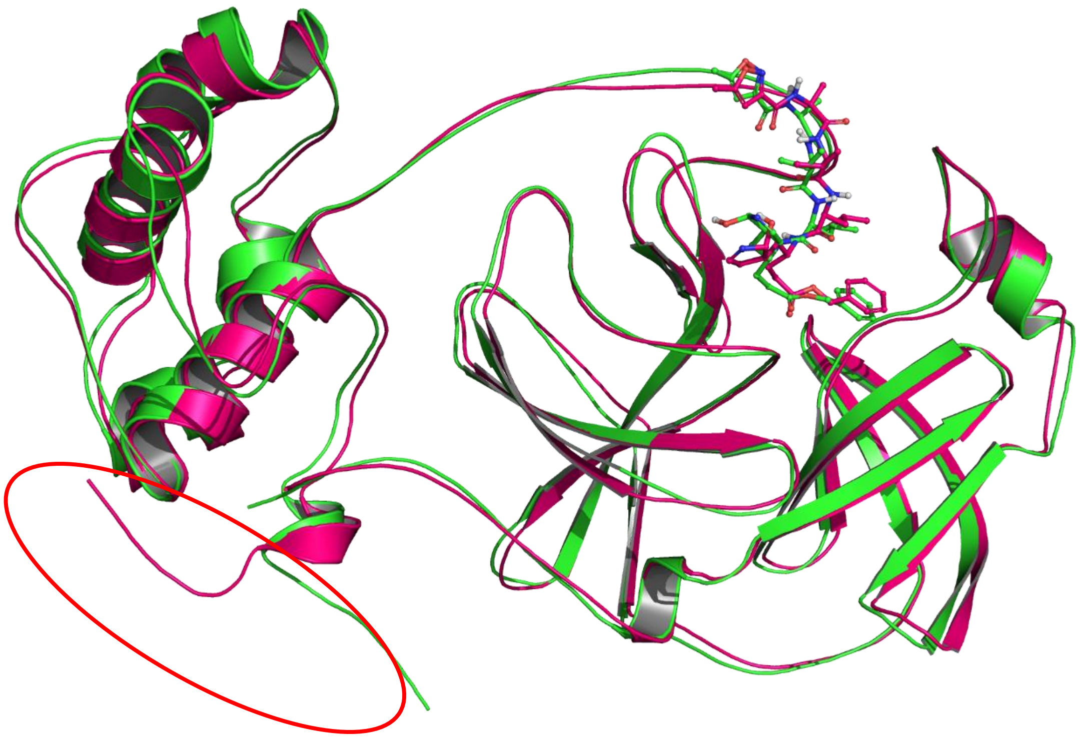

Figure SI 1: A snapshot of Tan-Mpro at 80 ns (purple) and 98 ns (green) of simulation time.

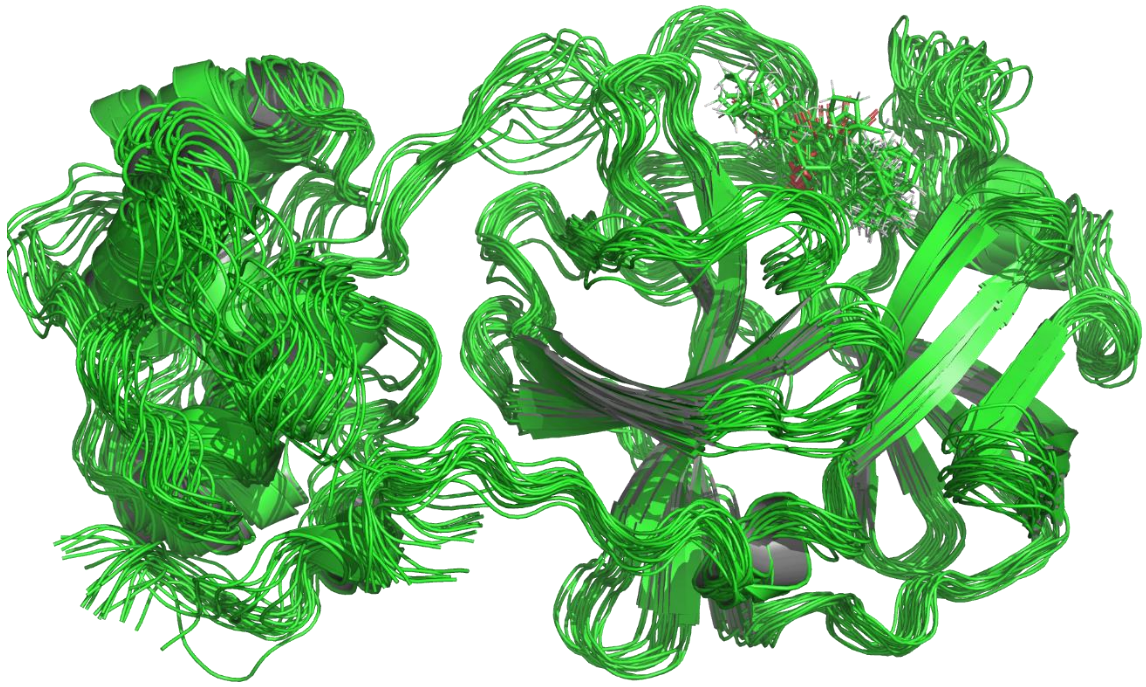

Figure SI 2: Snapshots of N3-Mpro during simulation time.

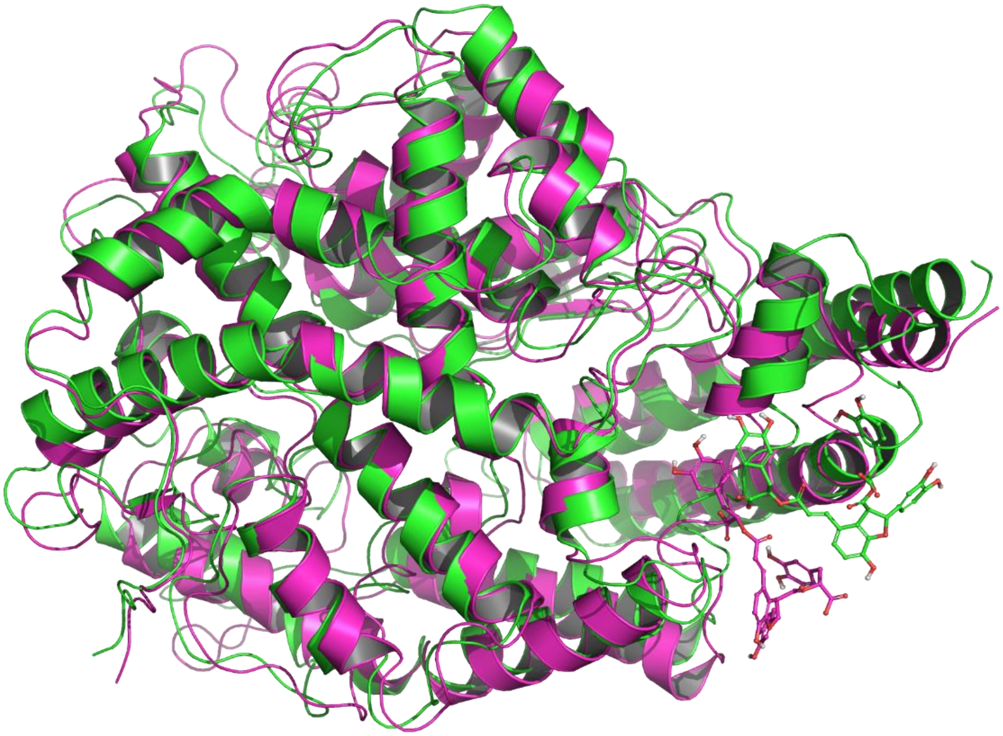

Figure SI 3: Snapshot of Sal-S at 0 ns (purple) and 100 ns (green).

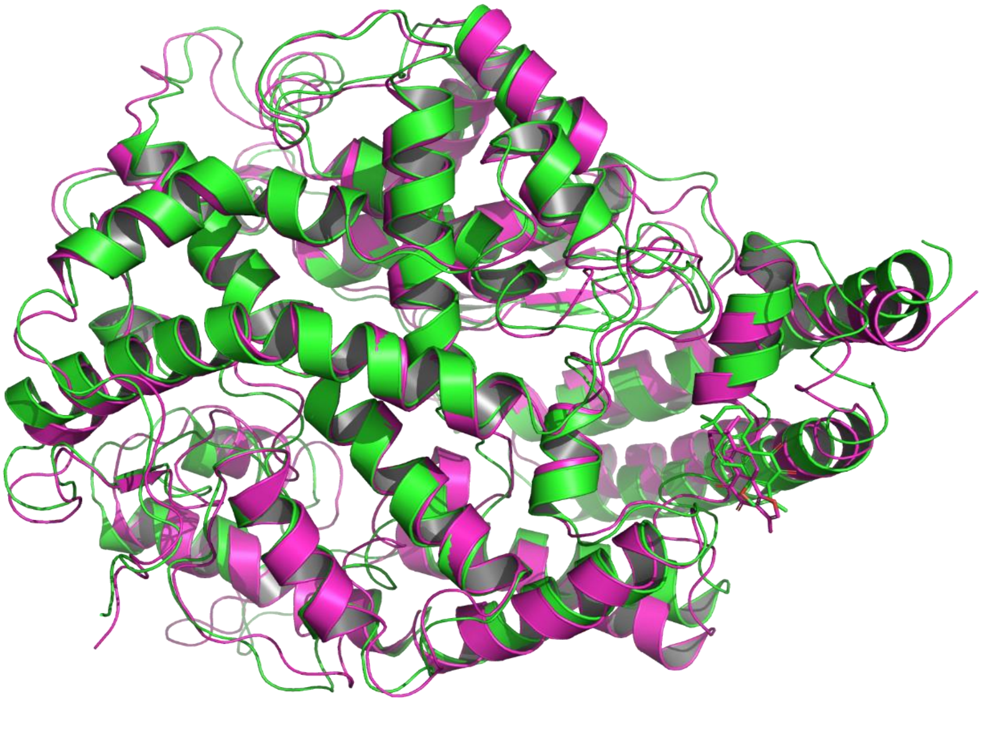

Figure SI 4: Snapshot of Tan-S at 0 ns (purple) and 100 ns (green).

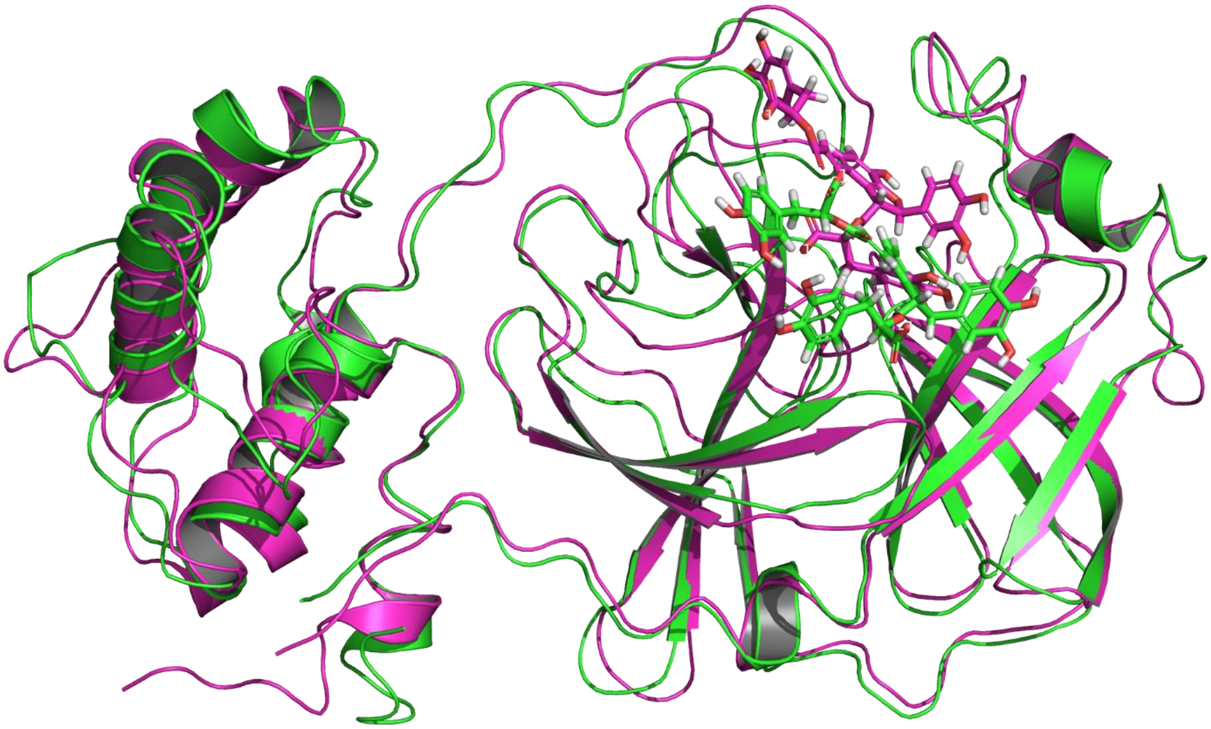

Figure SI 5: Snapshot of Sal-Mpro at 0 ns (purple) and 100 ns (green).

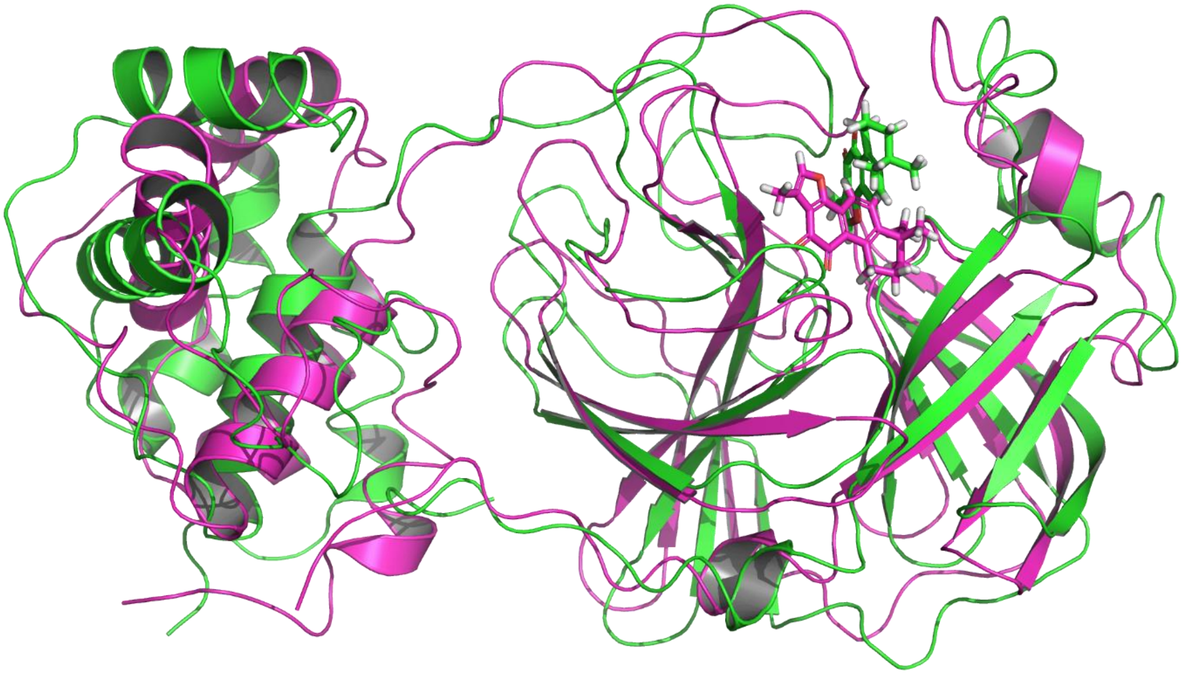

Figure SI 6: Snapshot of Tan-Mpro at 0 ns (purple) and 100 ns (green).

Table SI 1: Anti-viral activity against (SARS-CoV-2) (hCoV-19/Egypt/NRC-03/2020 (Accession Number on GSAID: EPI_ISL_430820) for the tested compounds (**1-6**) measured by Plaque reduction assay.

	**Sample**	**Conc.**	**Viral count after treatment**	**Virus control (PFU/ml)**	**Viral Inhibition %**
		**µg/ml**	**(PFU/ml)**		
**1**	***Tanshinone IIA***	50	0	10.8 X10^5	100
		25	0		100
		12.5	0		100
		6.25	0		100
**2**	***Carnosic acid***	50	8 X10^4	10.8 X10^5	92.5
		25	2.0 X10^5		81.4
		12.5	2.2 X10^5		79.6
		6.25	2.6 X10^5		75.9
**3**	***Rosmarinic acid***	50	3.1 X10^5	10.4 X10^5	70.2
		25	3.2 X10^5		69.2
		12.5	4.0 X10^5		61.5
		6.25	4.4 X10^5		57.6
**4**	***Salvianolic acid***	50	2.5 X10^5	9.0 X10^5	72
		25	4.2 X10^5		53.3
		12.5	4.4 X10^5		51.1
		6.25	5.3 X10^5		41.1
**5**	***Baicalein***	50	2.2 X10^5	7.8 X10^5	71.7
		25	2.6 X10^5		66.6
		12.5	2.8 X10^5		64.1
		6.25	3.5 X10^5		55
**6**	***Glycyrrhetinic acid***	50	3.1 X10^5	14.1 X10^5	78
		25	3.2 X10^5		77.3
		12.5	3.4 X10^5		75.8
		6.25	3.7 X10^5		73.7

Table SI 2: Plates of the anti-viral activity against (SARS-CoV-2) measured using plaque reduction assay for the tested compounds (**1-6**).

	**Tanshinone IIA**	**Carnosic acid**	**Rosmarinic acid**	**Salvianolic acid**	**Baicalein**	**Glycyrrhetinic acid**
**Virus control**	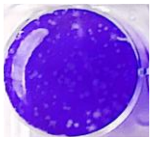	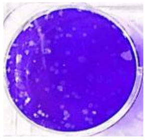	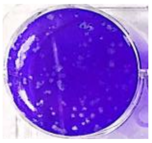	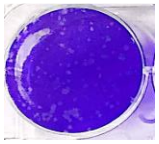	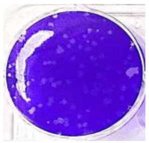	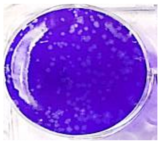
**0.012µg/µl**	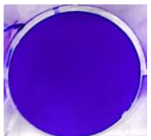	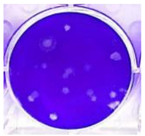	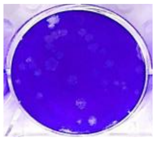	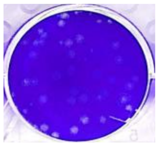	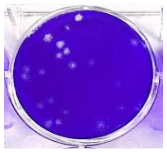	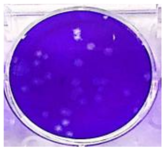
**0.025µg/µl**	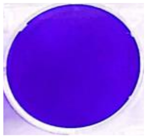	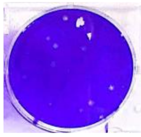	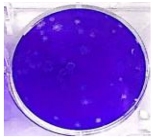	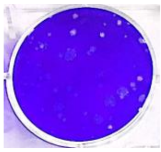	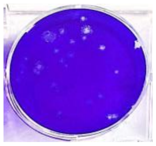	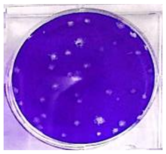
**0.05µg/µl**	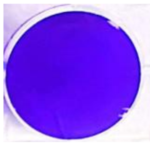	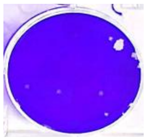	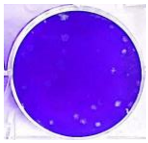	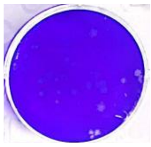	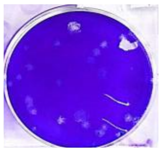	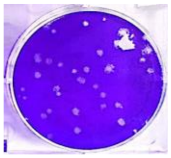
**0.006µg/µl**	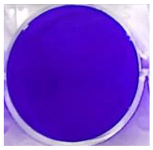	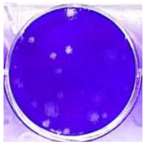	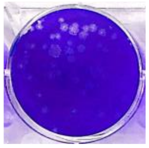	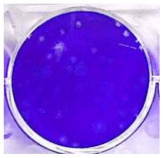	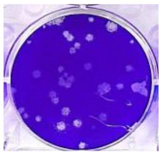	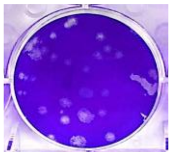
**Cell control**	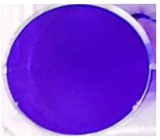	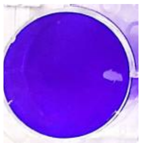	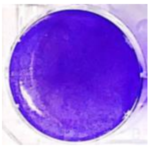	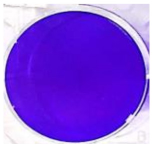	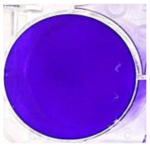	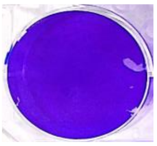

References

1. Release, S., 3: Desmond molecular dynamics system, DE Shaw research, New York, NY, 2017. *Maestro-Desmond Interoperability Tools, Schrödinger, New York, NY* **2017**.
2. Harder, E.; Damm, W.; Maple, J.; Wu, C.; Reboul, M.; Xiang, J. Y.; Wang, L.; Lupyan, D.; Dahlgren, M. K.; Knight, J. L., OPLS3: a force field providing broad coverage of drug-like small molecules and proteins. *Journal of chemical theory and computation* **2016,** *12* (1), 281-296.
3. Jorgensen, W. L.; Chandrasekhar, J.; Madura, J. D.; Impey, R. W.; Klein, M. L., Comparison of simple potential functions for simulating liquid water. *The Journal of chemical physics* **1983,** *79* (2), 926-935.
4. Neria, E.; Fischer, S.; Karplus, M., Simulation of activation free energies in molecular systems. *The Journal of chemical physics* **1996,** *105* (5), 1902-1921.
5. Manual, D. U., Desmond2. 2. **2009**.
6. Martyna, G. J.; Klein, M. L.; Tuckerman, M., Nosé–Hoover chains: The canonical ensemble via continuous dynamics. *The Journal of chemical physics* **1992,** *97* (4), 2635-2643.
7. Martyna, G. J.; Tobias, D. J.; Klein, M. L., Constant pressure molecular dynamics algorithms. *The Journal of chemical physics* **1994,** *101* (5), 4177-4189.
